# Supplementary material for: Registered report: Stress testing predictive models of ideological prejudice
Source: PLoS One. 2025 Oct 13;20(10):e0334152. doi: 10.1371/journal.pone.0334152 (PMC12517488; doi:10.1371/journal.pone.0334152)
Supplement: S1 Appendix — This supplemental file includes feeling thermometers, status rating, ideology rating, and choice rating items for the new data collection described in the manuscript and the results. (DOCX) [file pone.0334152.s001.docx]

**S1 Appendix**

**Group Ratings Survey**

**Directions:** In the present study we will ask you to rate your perceptions of a variety of social groups. We are interested in people’s opinions about these groups. On the following pages we will give you the names of groups and questions about them. Please read each statement relating to each group and use the scales provided to indicate your opinions. Please click the forward button to continue.

**Please rate your feelings toward the following groups, from 0 (cold/unfavorable) to 100 (warm/favorable).**

| Gay people | 0                50 100 cold                 neutral                            warm |
| --- | --- |
| Straight people | 0                50 100 cold                 neutral                            warm |
| Non-profits | 0                50 100 cold                 neutral                            warm |
| Corporations | 0                50 100 cold                 neutral                            warm |
| Labor | 0                50 100 cold                 neutral                            warm |
| Management | 0                50 100 cold                 neutral                            warm |
| Foreign people | 0                50 100 cold                 neutral                            warm |
| Local people | 0                50 100 cold                 neutral                            warm |
| Black people | 0                50 100 cold                 neutral                            warm |
| White people | 0                50 100 cold                 neutral                            warm |
| Mothers | 0                50 100 cold                 neutral                            warm |
| Fathers | 0                50 100 cold                 neutral                            warm |
| Democrats | 0                50 100 cold                 neutral                            warm |
| Republicans | 0                50 100 cold                 neutral                            warm |
| Liberals | 0                50 100 cold                 neutral                            warm |
| Conservatives | 0                50 100 cold                 neutral                            warm |
| Religious people | 0                50 100 cold                 neutral                            warm |
| Atheists | 0                50 100 cold                 neutral                            warm |
| Scientists | 0                50 100 cold                 neutral                            warm |
| Capitalists | 0                50 100 cold                 neutral                            warm |
| Socialists | 0                50 100 cold                 neutral                            warm |

**Please read the following statement and use the slider below to indicate your response.**

Some groups in society have higher status. That is, they have more education, they have more prestigious jobs, and they are more economically successful than other groups. Some groups have lower status. That is, they have less education, less prestigious jobs, and are less economically successful than other groups. And, of course, some groups are more in the middle.

Where would you place the following groups in terms of prestigious jobs, economic success, and overall status?

| Gay people | 0                50 100 low status middle high status |
| --- | --- |
| Straight people | 0                50 100 low status middle high status |
| Non-profits | 0                50 100 low status middle high status |
| Corporations | 0                50 100 low status middle high status |
| Labor | 0                50 100 low status middle high status |
| Management | 0                50 100 low status middle high status |
| Foreign people | 0                50 100 low status middle high status |
| Local people | 0                50 100 low status middle high status |
| Black people | 0                50 100 low status middle high status |
| White people | 0                50 100 low status middle high status |
| Mothers | 0                50 100 low status middle high status |
| Fathers | 0                50 100 low status middle high status |
| Democrats | 0                50 100 low status middle high status |
| Republicans | 0                50 100 low status middle high status |
| Liberals | 0                50 100 low status middle high status |
| Conservatives | 0                50 100 low status middle high status |
| Religious people | 0                50 100 low status middle high status |
| Atheists | 0                50 100 low status middle high status |
| Scientists | 0                50 100 low status middle high status |
| Capitalists | 0                50 100 low status middle high status |
| Socialists | 0                50 100 low status middle high status |

**Please read the following statement and use the slider below to indicate your response.**

For each group indicate whether you think the group is typically a liberal or conservative group.

| Gay people | 0                25 50 75 100 very liberal liberal moderate conservative very conservative |
| --- | --- |
| Straight people | 0                25 50 75 100 very liberal liberal moderate conservative very conservative |
| Non-profits | 0                25 50 75 100 very liberal liberal moderate conservative very conservative |
| Corporations | 0                25 50 75 100 very liberal liberal moderate conservative very conservative |
| Labor | 0                25 50 75 100 very liberal liberal moderate conservative very conservative |
| Management | 0                25 50 75 100 very liberal liberal moderate conservative very conservative |
| Foreign people | 0                25 50 75 100 very liberal liberal moderate conservative very conservative |
| Local people | 0                25 50 75 100 very liberal liberal moderate conservative very conservative |
| Black people | 0                25 50 75 100 very liberal liberal moderate conservative very conservative |
| White people | 0                25 50 75 100 very liberal liberal moderate conservative very conservative |
| Mothers | 0                25 50 75 100 very liberal liberal moderate conservative very conservative |
| Fathers | 0                25 50 75 100 very liberal liberal moderate conservative very conservative |
| Democrats | 0                25 50 75 100 very liberal liberal moderate conservative very conservative |
| Republicans | 0                25 50 75 100 very liberal liberal moderate conservative very conservative |
| Liberals | 0                25 50 75 100 very liberal liberal moderate conservative very conservative |
| Conservatives | 0                25 50 75 100 very liberal liberal moderate conservative very conservative |
| Religious people | 0                25 50 75 100 very liberal liberal moderate conservative very conservative |
| Atheists | 0                25 50 75 100 very liberal liberal moderate conservative very conservative |
| Scientists | 0                25 50 75 100 very liberal liberal moderate conservative very conservative |
| Capitalists | 0                25 50 75 100 very liberal liberal moderate conservative very conservative |
| Socialists | 0                25 50 75 100 very liberal liberal moderate conservative very conservative |

**Please read the following statement and use the slider below to indicate your response.**

Sometimes people have choice and control of whether they belong to a particular group. Other times, they do not have much choice and control over whether they belong to a particular group.

To what extent can members of this group choose or control whether they actually belong to this group?

| Gay people | 0                25 50 75 100 not at all slightly somewhat much very much |
| --- | --- |
| Straight people | 0                25 50 75 100 not at all slightly somewhat much very much |
| Non-profits | 0                25 50 75 100 not at all slightly somewhat much very much |
| Corporations | 0                25 50 75 100 not at all slightly somewhat much very much |
| Labor | 0                25 50 75 100 not at all slightly somewhat much very much |
| Management | 0                25 50 75 100 not at all slightly somewhat much very much |
| Foreign people | 0                25 50 75 100 not at all slightly somewhat much very much |
| Local people | 0                25 50 75 100 not at all slightly somewhat much very much |
| Black people | 0                25 50 75 100 not at all slightly somewhat much very much |
| White people | 0                25 50 75 100 not at all slightly somewhat much very much |
| Mothers | 0                25 50 75 100 not at all slightly somewhat much very much |
| Fathers | 0                25 50 75 100 not at all slightly somewhat much very much |
| Democrats | 0                25 50 75 100 not at all slightly somewhat much very much |
| Republicans | 0                25 50 75 100 not at all slightly somewhat much very much |
| Liberals | 0                25 50 75 100 not at all slightly somewhat much very much |
| Conservatives | 0                25 50 75 100 not at all slightly somewhat much very much |
| Religious people | 0                25 50 75 100 not at all slightly somewhat much very much |
| Atheists | 0                25 50 75 100 not at all slightly somewhat much very much |
| Scientists | 0                25 50 75 100 not at all slightly somewhat much very much |
| Capitalists | 0                25 50 75 100 not at all slightly somewhat much very much |
| Socialists | 0                25 50 75 100 not at all slightly somewhat much very much |

**Group Ratings Survey Results**

| **Group Name** | **Ideology** | **Status** | **Choice** |
| --- | --- | --- | --- |
| Black | 36.12 | 47.37 | 27.67 |
| White | 60.67 | 67.56 | 32.45 |
| Gay | 18.25 | 49.96 | 34.62 |
| Straight | 59.86 | 63.77 | 43.34 |
| Mother | 47.21 | 55.36 | 61.31 |
| Father | 60.14 | 63.50 | 65.22 |
| Foreign | 46.01 | 47.13 | 32.03 |
| Local | 56.79 | 53.07 | 48.66 |
| Non-profits | 37.65 | 54.33 | 72.34 |
| Corporations | 71.84 | 82.01 | 74.74 |
| Labor | 50.19 | 45.02 | 60.54 |
| Management | 67.32 | 72.95 | 72.81 |
| Democrats | 24.15 | 58.50 | 80.92 |
| Republicans | 86.36 | 60.53 | 79.62 |
| Liberal | 14.09 | 57.64 | 77.19 |
| Conservative | 91.70 | 61.50 | 79.10 |
| Religious | 78.52 | 56.38 | 73.20 |
| Atheist | 29.23 | 52.35 | 72.21 |
| Scientists | 38.30 | 75.75 | 77.13 |
| Capitalists | 73.51 | 73.90 | 74.65 |
| Socialists | 26.16 | 46.20 | 76.04 |
