# Supplementary figures and images for: Registered report: Stress testing predictive models of ideological prejudice
Source: PLoS One. 2025 Oct 13;20(10):e0334152. doi: 10.1371/journal.pone.0334152 (PMC12517488; doi:10.1371/journal.pone.0334152)

**S2 Appendix**

**Model x Measure Interaction Post Hoc Results (see lines 1486 – 1539 in the Study 1 code)**


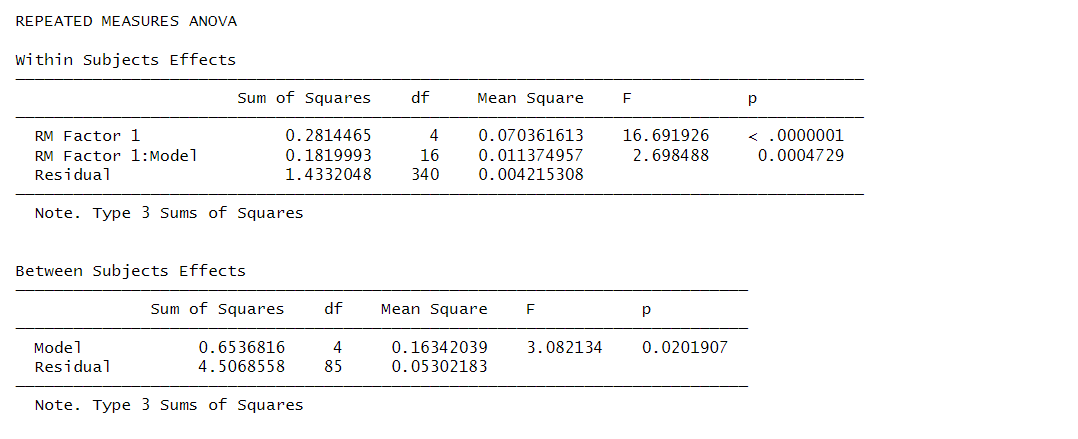


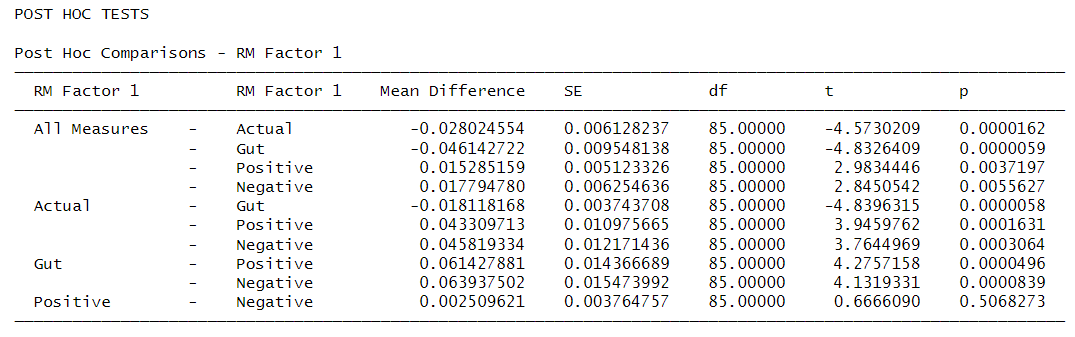


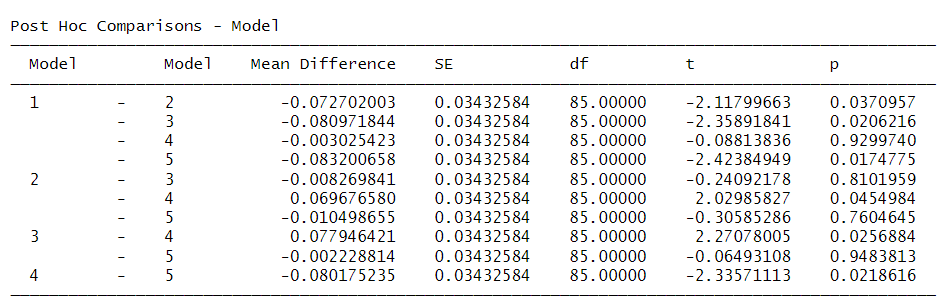


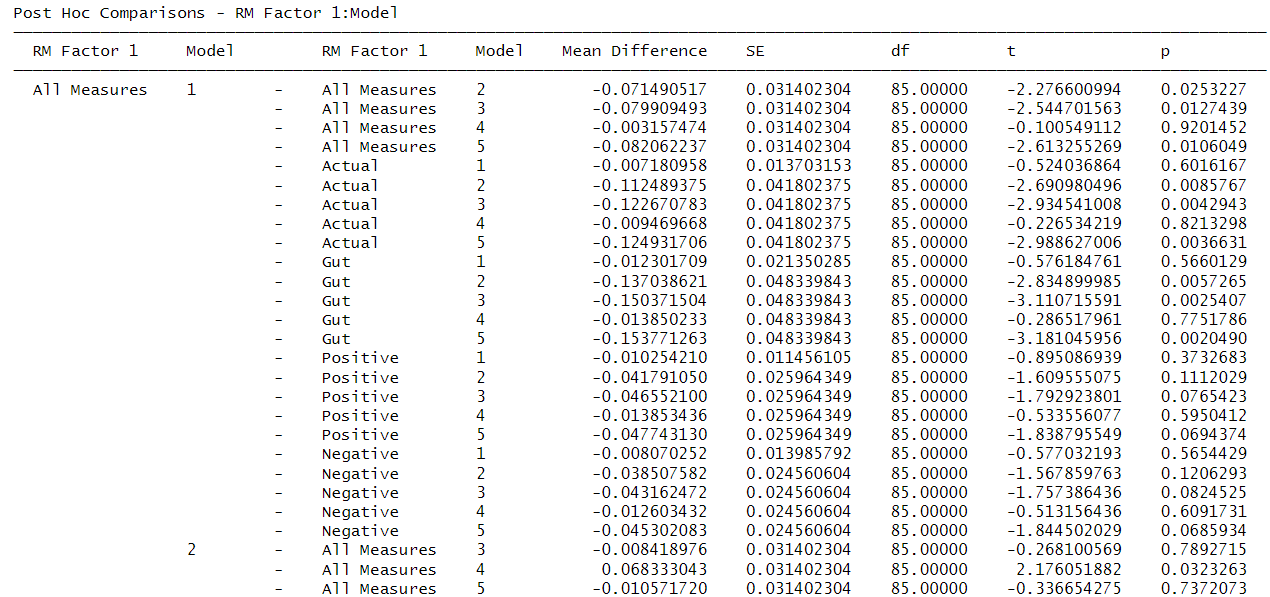


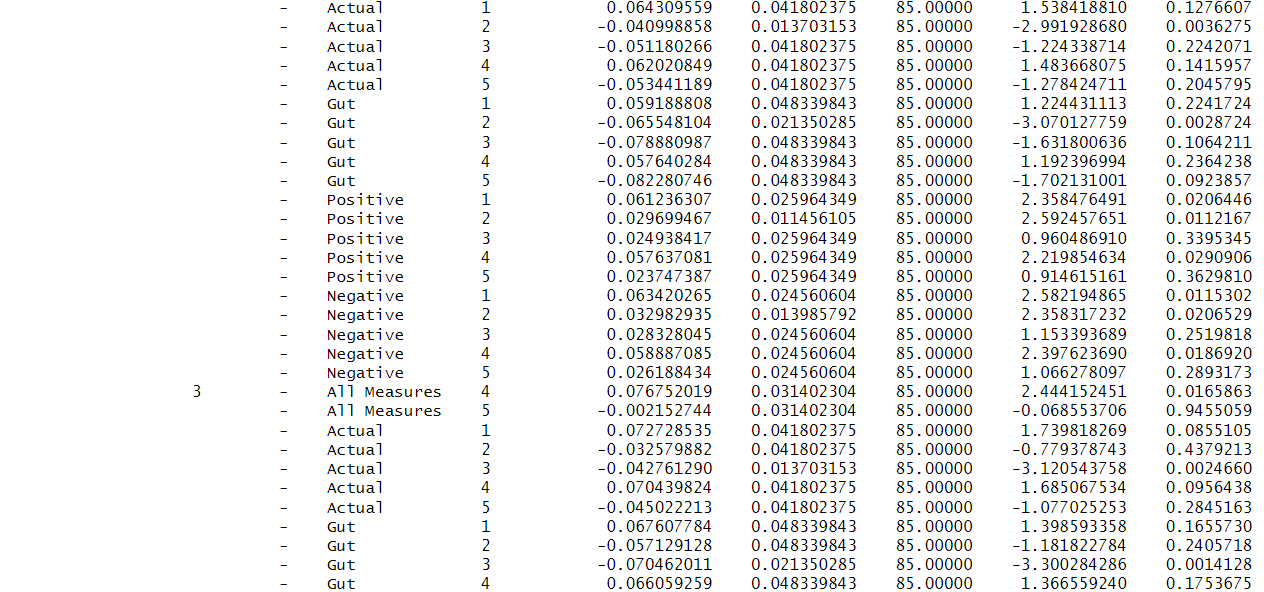


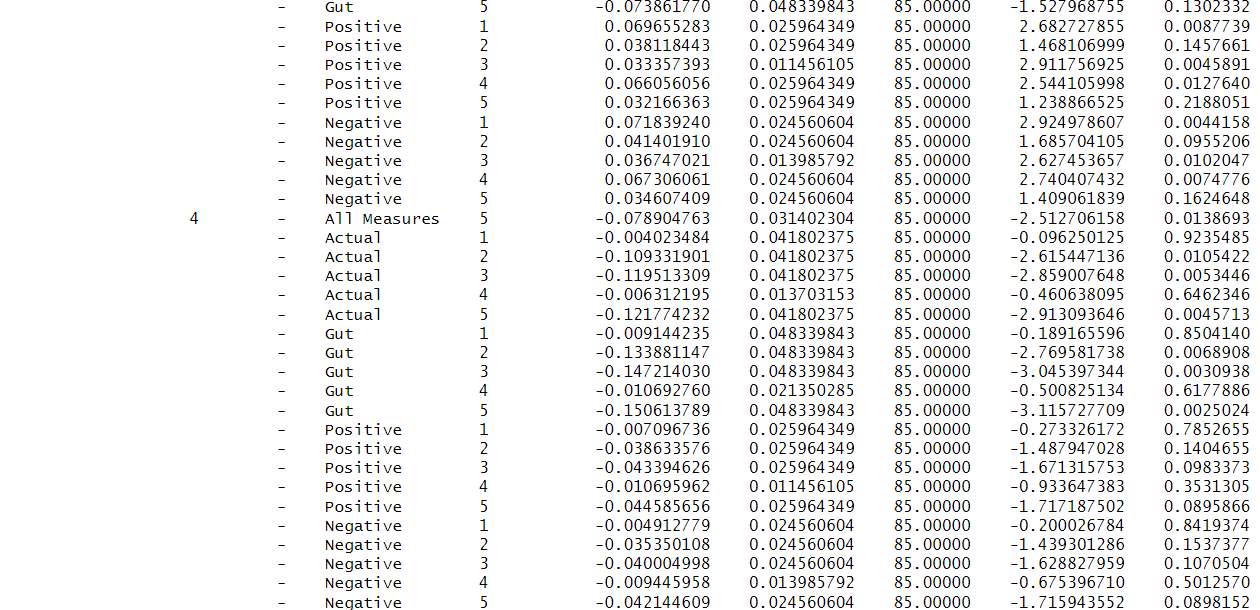


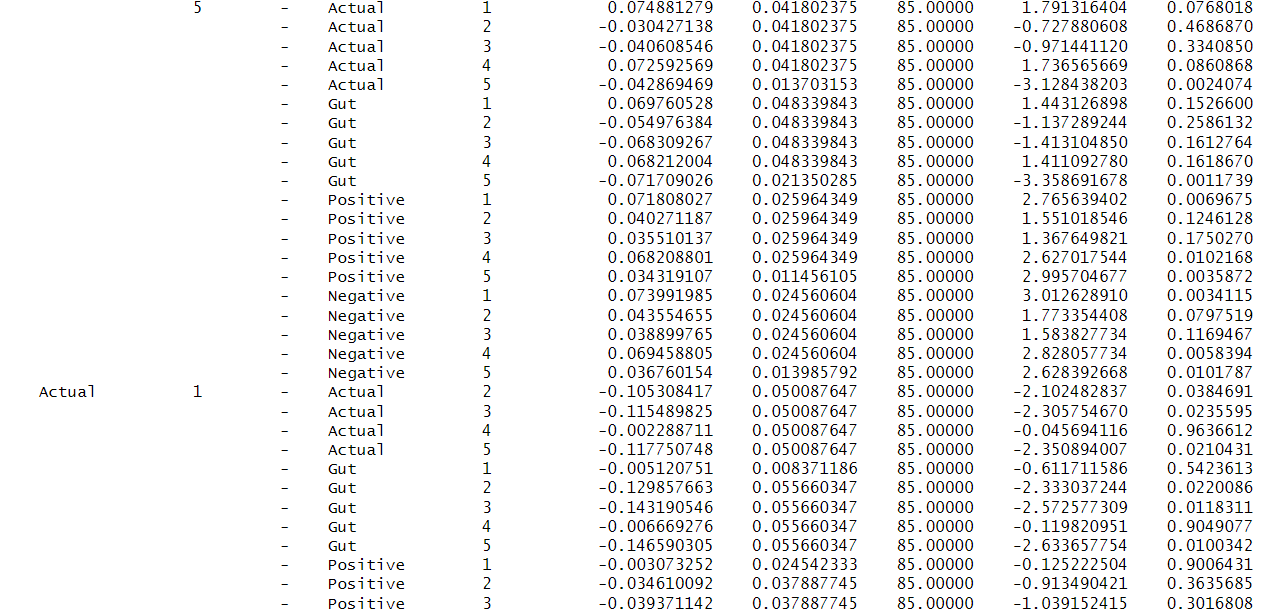


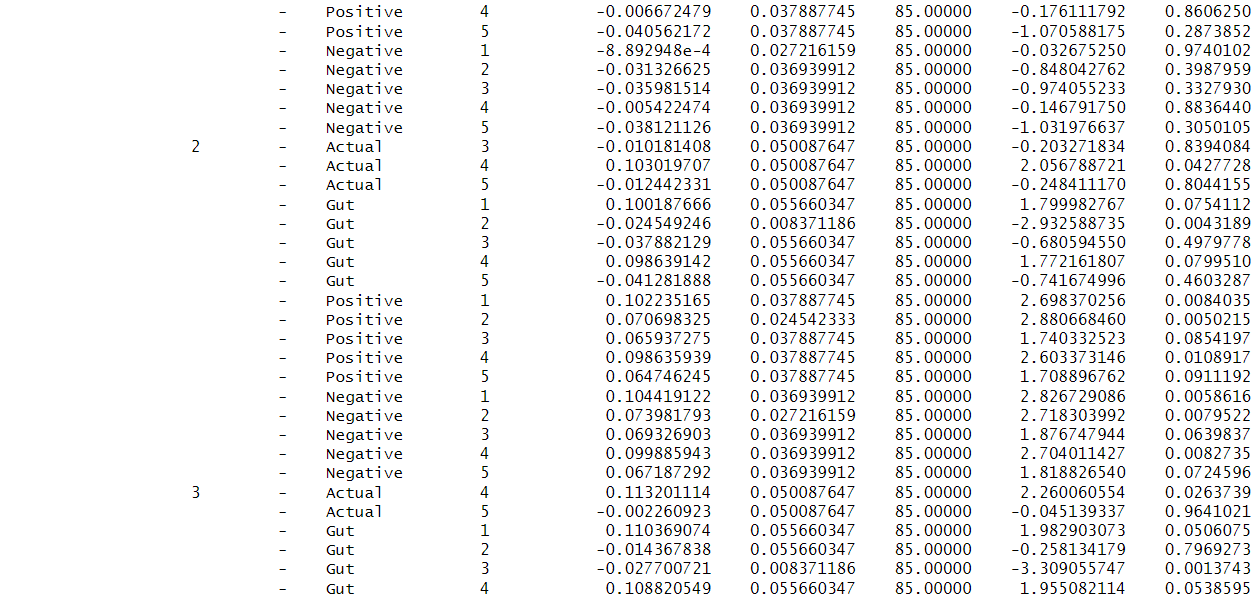


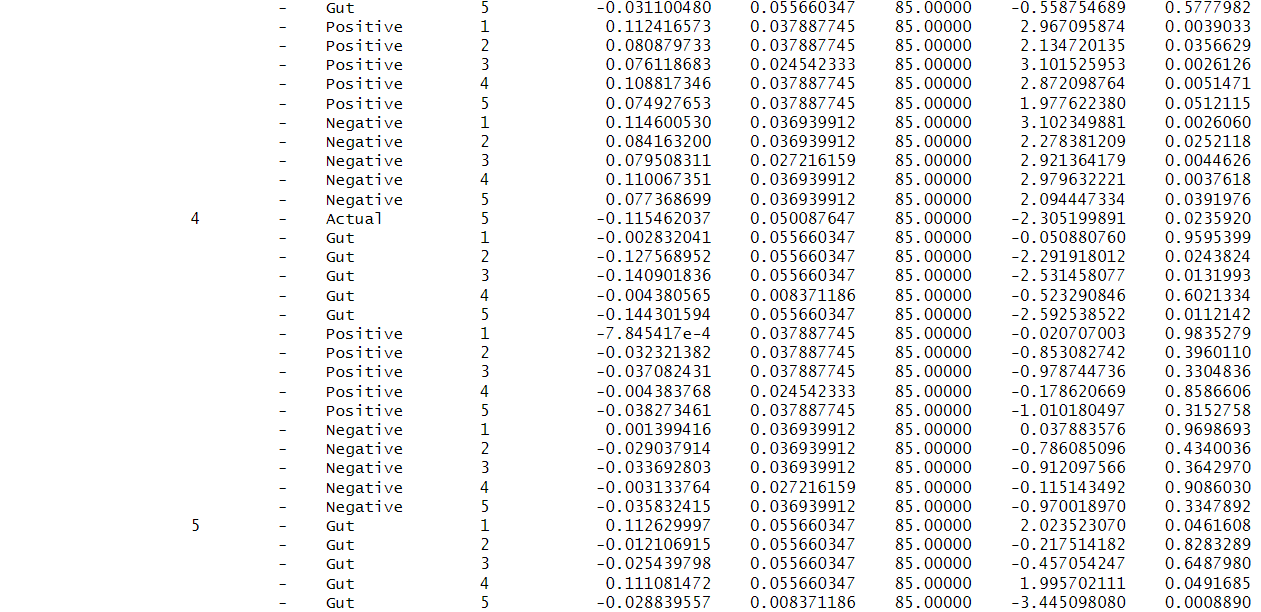


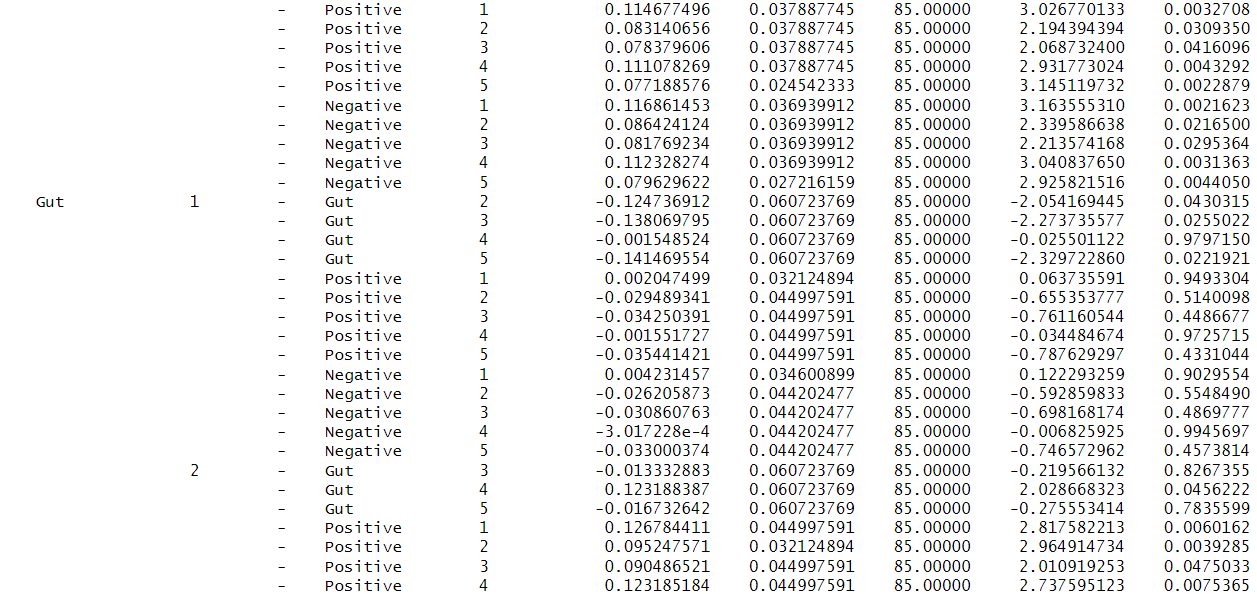


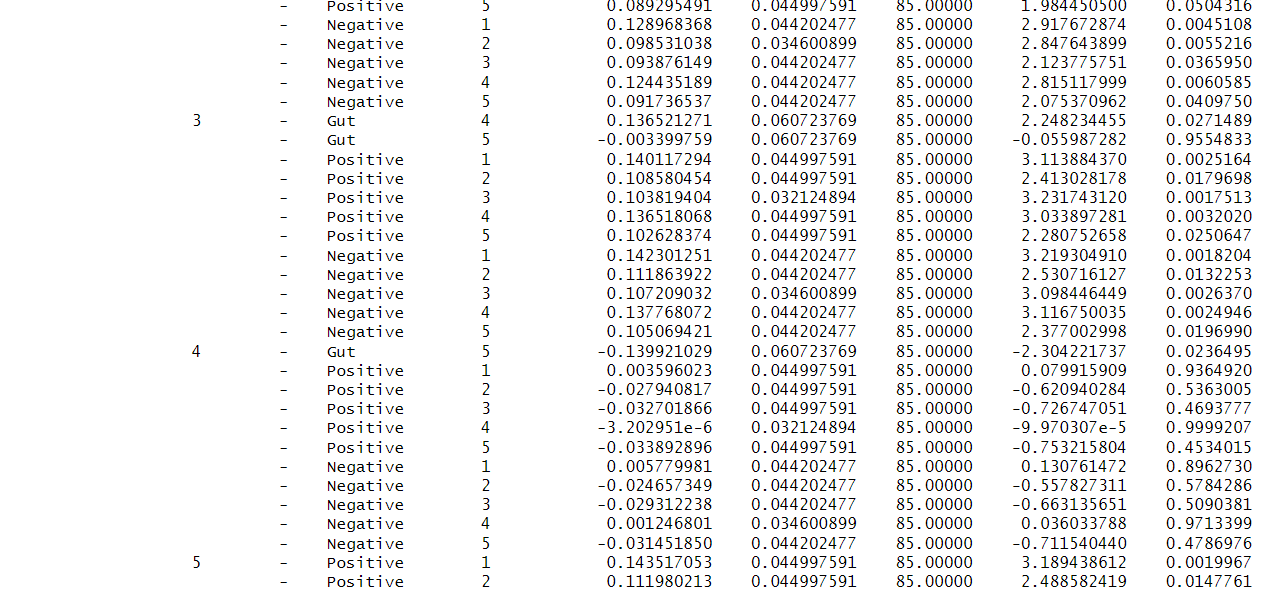


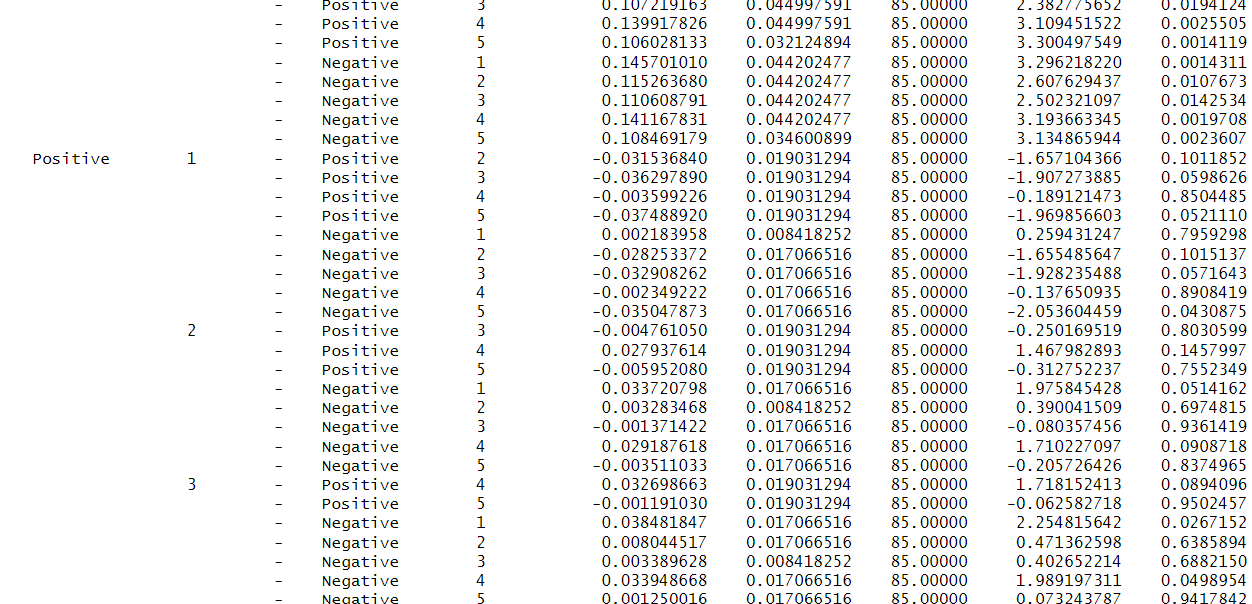


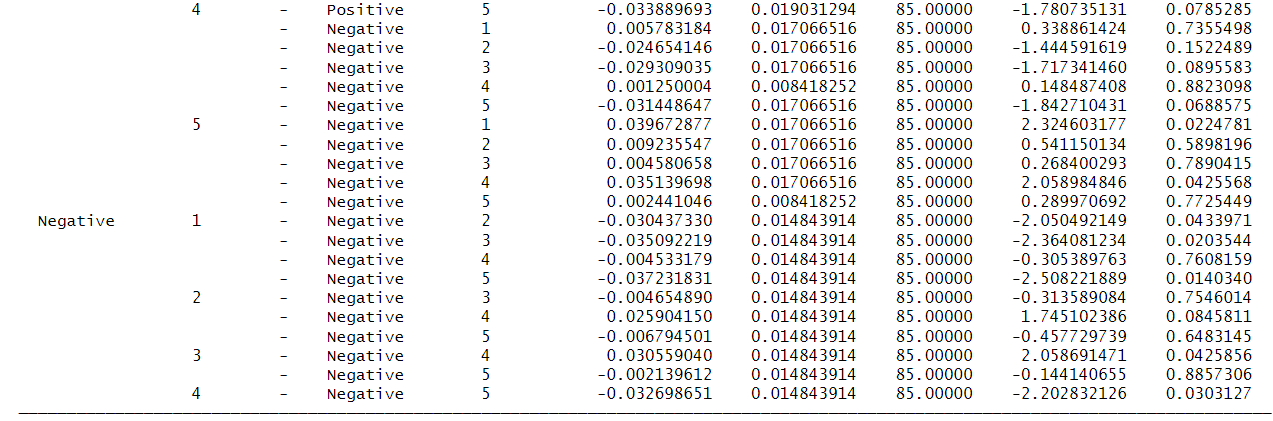

Supplement: S2 Appendix — This supplemental file includes the post hoc tests examining the Model x Measure interaction in the Mixed ANOVA. (DOCX) [file pone.0334152.s002.docx]
