# Supplementary material for: Registered report: Stress testing predictive models of ideological prejudice
Source: PLoS One. 2025 Oct 13;20(10):e0334152. doi: 10.1371/journal.pone.0334152 (PMC12517488; doi:10.1371/journal.pone.0334152)
Supplement: S3 Appendix — This supplemental file includes the output for the Study 1 and Study 2 analyses, with demographic controls removed from the multilevel models. (DOCX) [file pone.0334152.s003.docx]

**S3 Appendix**

**Table S3.1 Post Hoc Tests for Absolute Prejudice Measures with Controls Removed.**

| Comparison | |  |  |  |  |
| --- | --- | --- | --- | --- | --- |
| Prejudice Measures | Mean Difference | *SE* | *df* | *t* | *p* |
| All Measures – Actual | -0.03 | 0.01 | 85 | -5.12 | <.001 |
| All Measures - Gut | -0.05 | 0.01 | 85 | -5.56 | <.001 |
| All Measures - Positive | 0.02 | 0.01 | 85 | 3.94 | <.001 |
| All Measures - Negative | 0.02 | 0.01 | 85 | 3.50 | <.001 |
| Actual – Gut | -0.02 | 0.004 | 85 | -5.55 | <.001 |
| Actual – Positive | 0.06 | 0.01 | 85 | 4.72 | <.001 |
| Actual – Negative | 0.06 | 0.01 | 85 | 4.43 | <.001 |
| Gut – Positive | 0.07 | 0.01 | 85 | 5.07 | <.001 |
| Gut – Negative | 0.08 | 0.02 | 85 | 4.91 | <.001 |
| Positive – Negative | 0.001 | 0.004 | 85 | 0.36 | .721 |

Table S3.1 contains the mean differences for the main effect of the type of absolute prejudice measure. Compare this to Table 5 in the manuscript.

**Table S3.2**. **Post Hoc Tests for Model Comparisons with Controls Removed.**

| Comparison | |  |  |  |  |
| --- | --- | --- | --- | --- | --- |
| Model | Mean Difference | *SE* | *df* | *t* | *p* |
| Ideology – Status | -0.09 | 0.04 | 85 | -2.51 | .014 |
| Ideology – Choice | -0.10 | 0.04 | 85 | -2.77 | .007 |
| Ideology – Ideology + Status + Choice | -0.002 | 0.04 | 85 | -0.06 | .949 |
| Ideology – Null | -0.11 | 0.04 | 85 | -2.87 | .005 |
| Status – Choice | -0.01 | 0.04 | 85 | -0.26 | .794 |
| Status – Ideology + Status + Choice | 0.09 | 0.04 | 85 | 2.45 | .016 |
| Status – Null | -0.01 | 0.04 | 85 | -0.36 | .722 |
| Choice – Ideology + Status + Choice | 0.10 | 0.04 | 85 | 2.71 | .008 |
| Choice – Null | -0.004 | 0.04 | 85 | -0.09 | .925 |
| Ideology + Status + Choice – Null | -0.11 | 0.04 | 85 | -2.80 | .006 |

Table S3.2 contains the mean differences for the main effect of model type. Compare this to Table 6 in the manuscript.

**Table S3.3 Model x Measure Interaction Post Hoc Results**


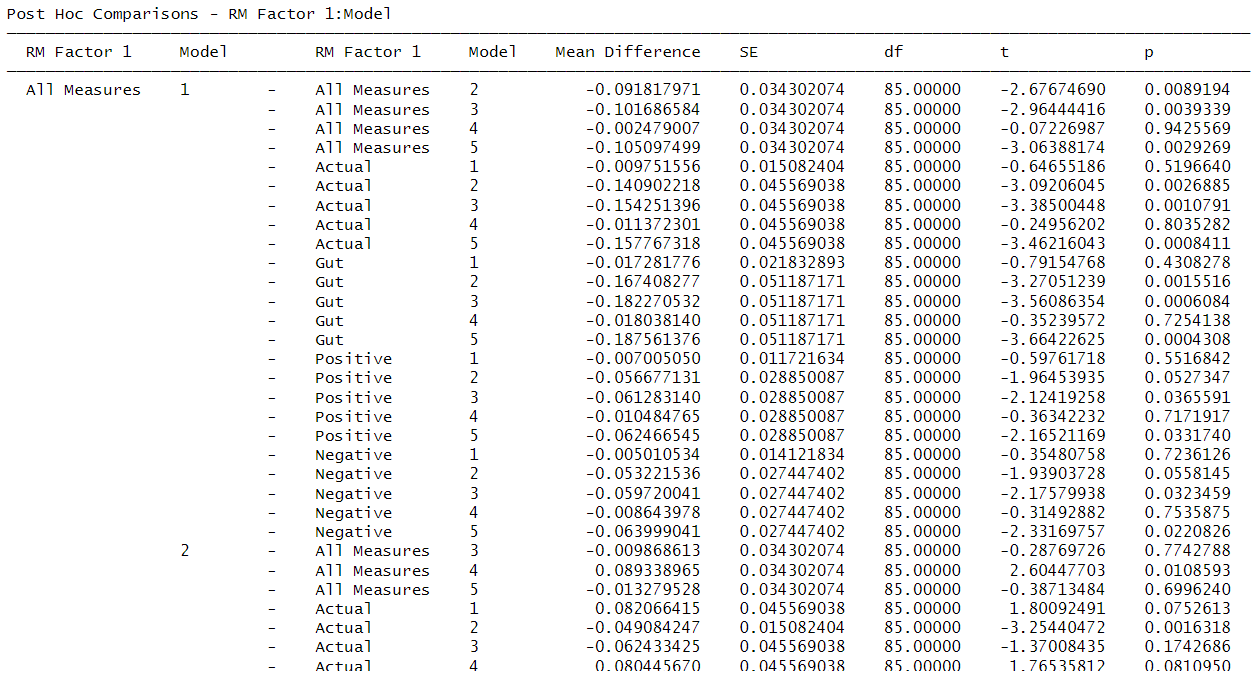


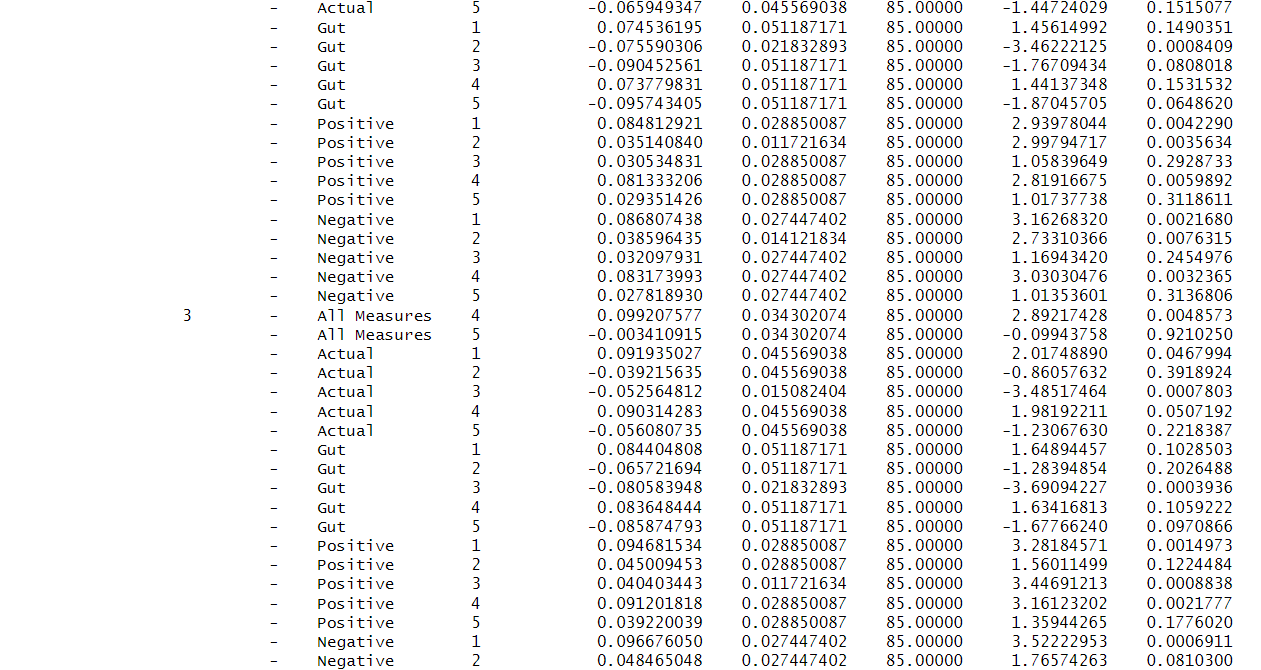


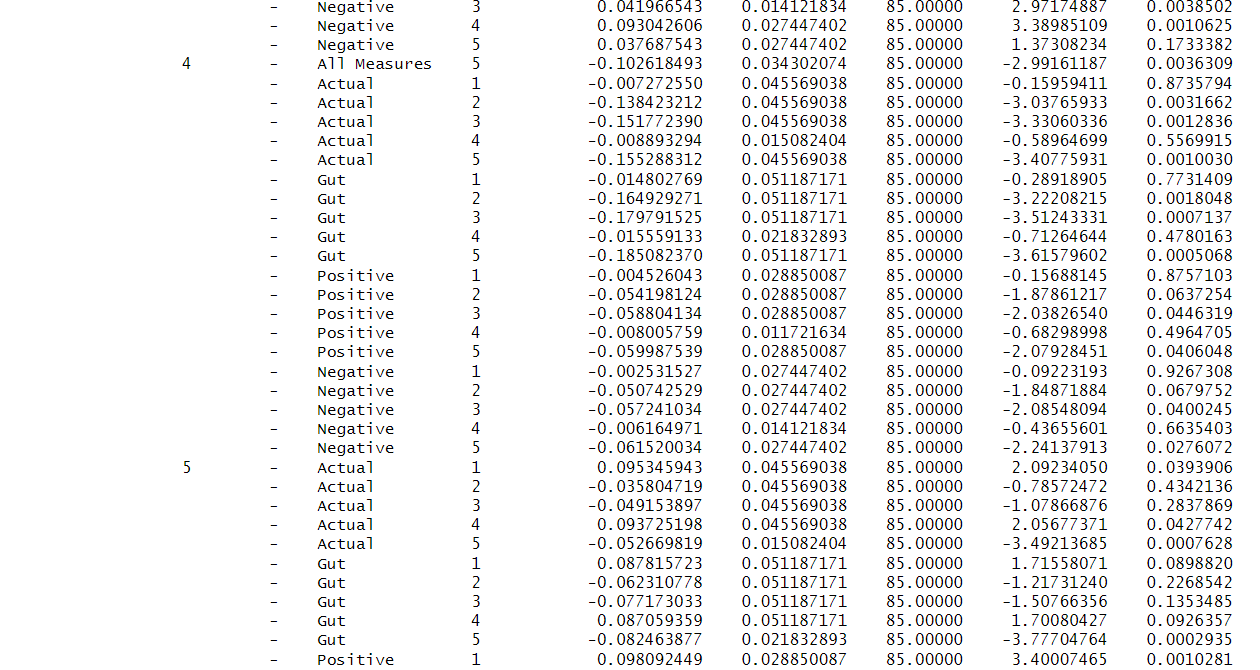

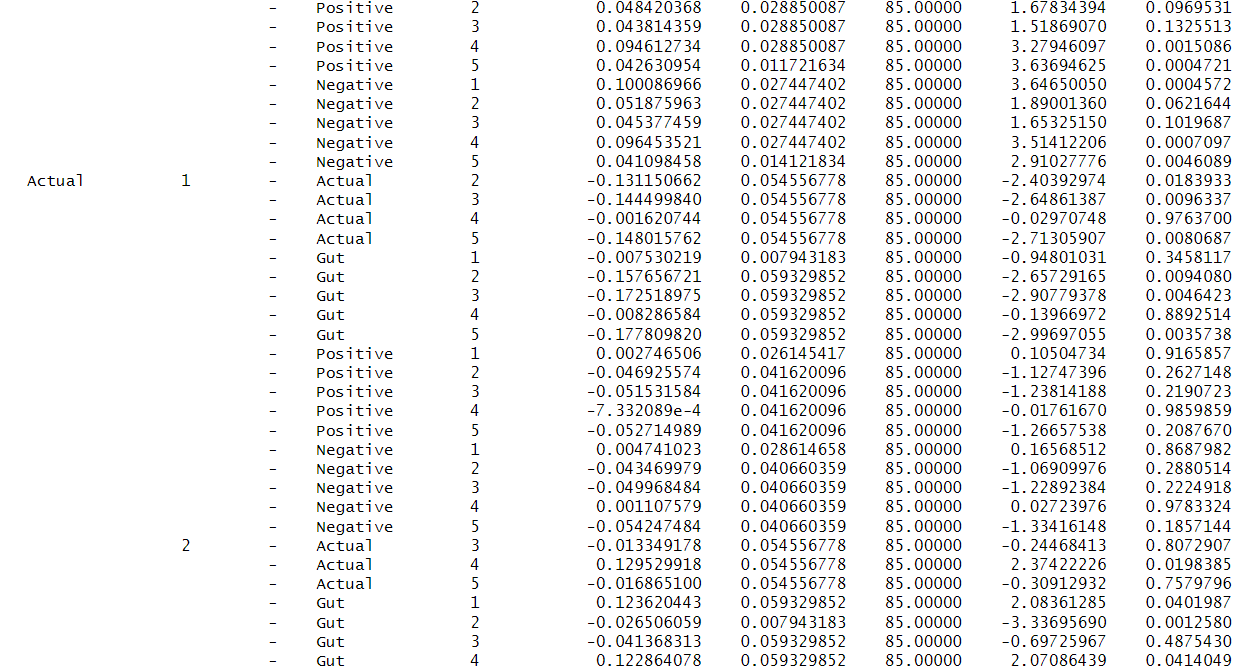

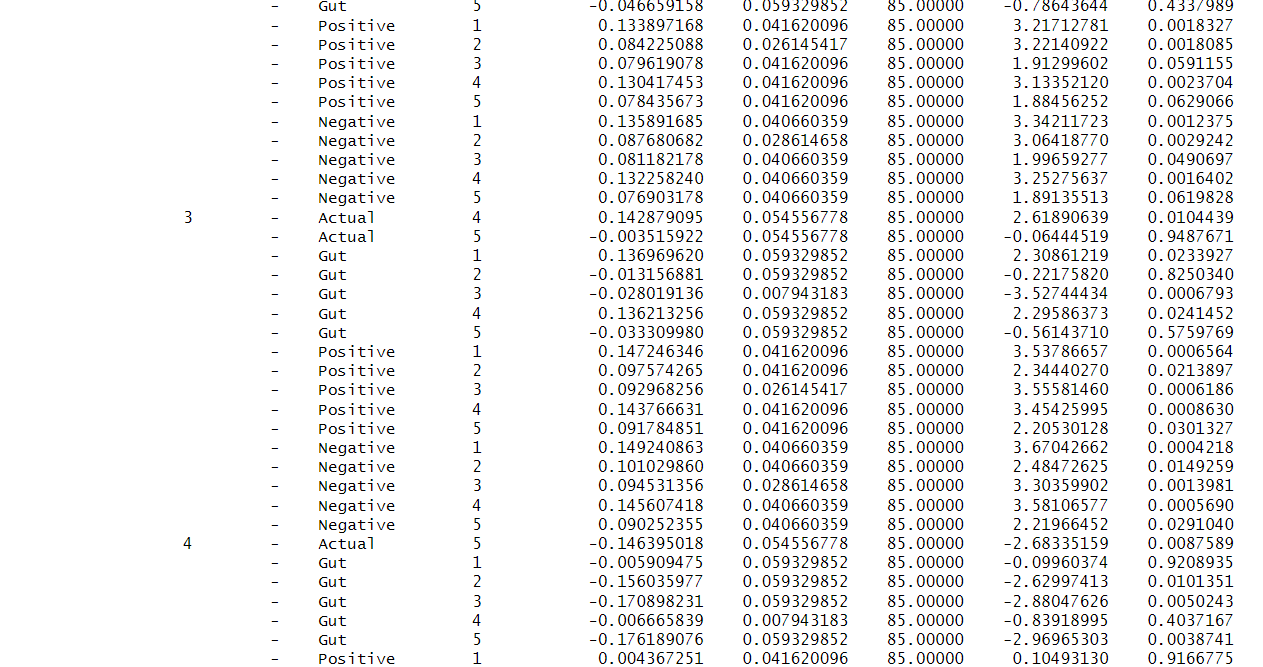

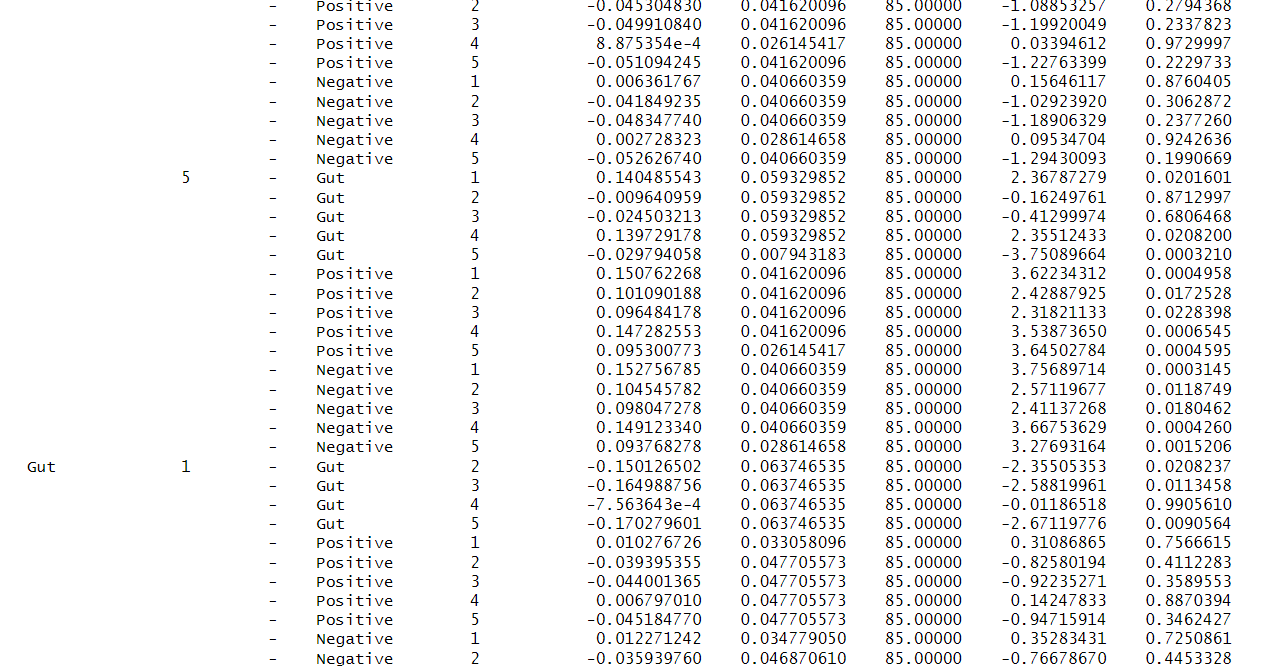


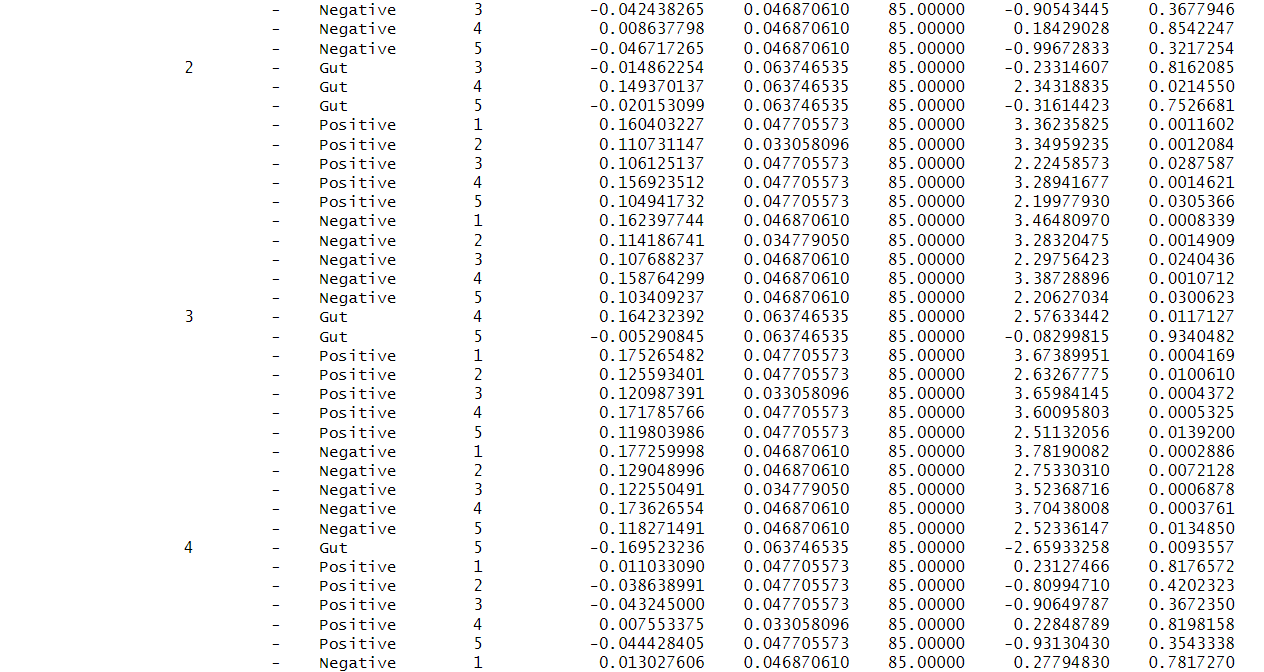

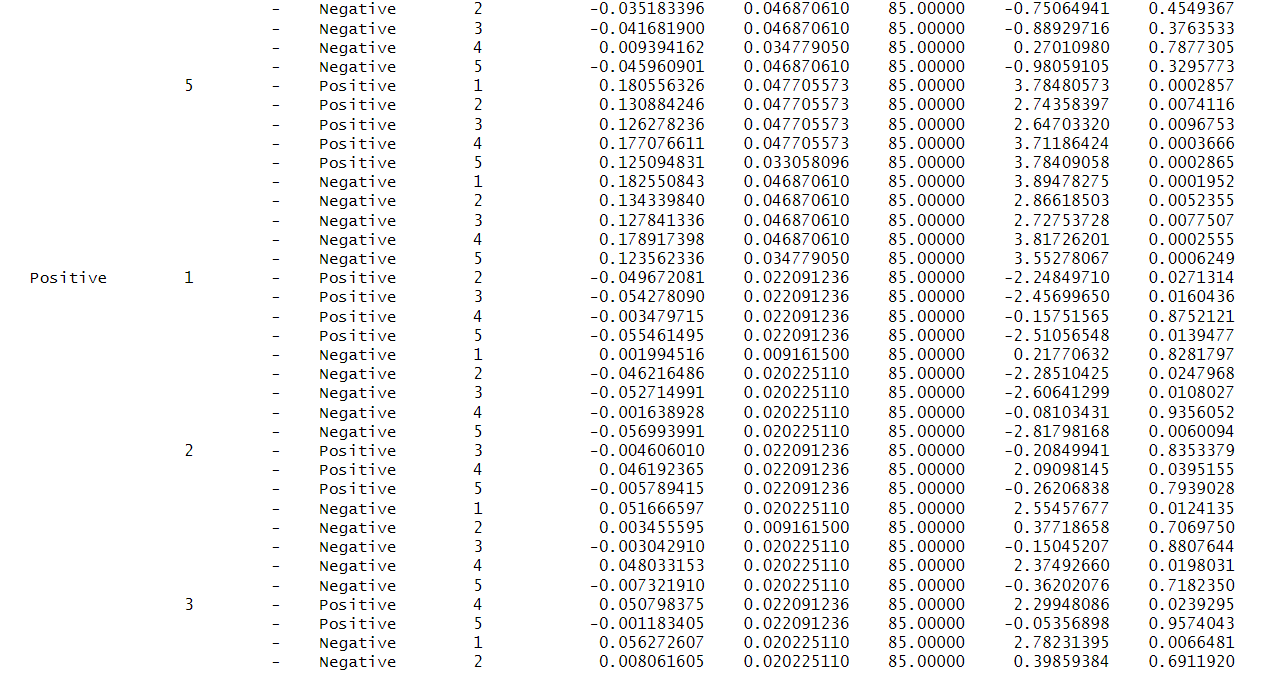

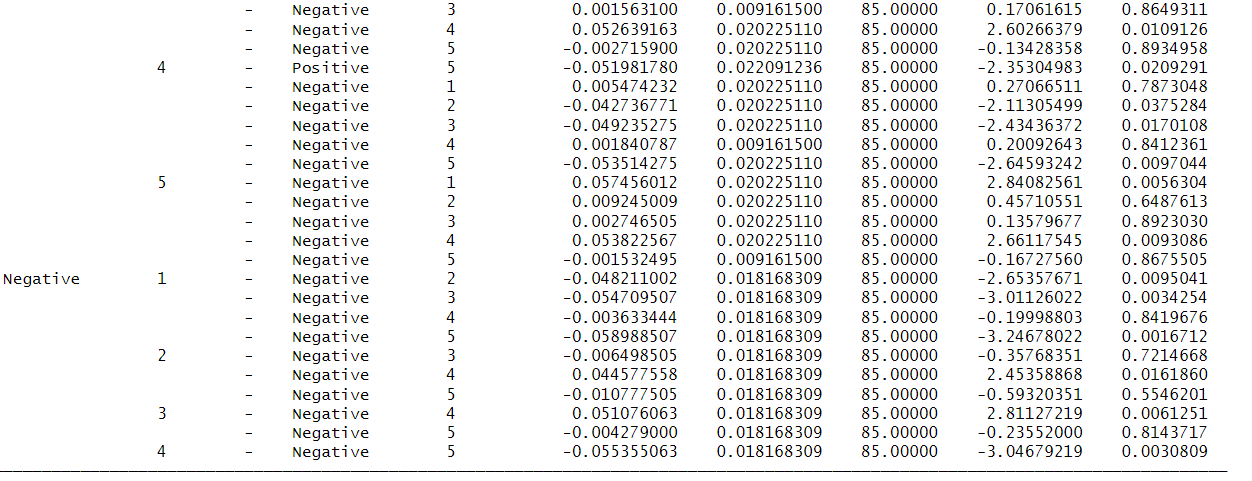


**Table S3.4. Post Hoc Tests for Relative Prejudice Measures.**

| Comparison | |  |  |  |  |
| --- | --- | --- | --- | --- | --- |
| Prejudice Measures | Mean Difference | *SE* | *df* | *t* | *p* |
| All Measures – Actual | -0.01 | 0.01 | 25 | -1.47 | .155 |
| All Measures - Gut | -0.01 | 0.01 | 25 | -1.35 | .189 |
| All Measures - Positive | -0.01 | 0.01 | 25 | -0.90 | .374 |
| All Measures - Negative | -0.003 | 0.01 | 25 | -0.65 | .520 |
| All Measures - Preference | -0.01 | 0.01 | 25 | -2.45 | .022 |
| All Measures – D-Score | -0.003 | 0.01 | 25 | -0.60 | .553 |
| Actual – Gut | 0.001 | 0.01 | 25 | -0.11 | .916 |
| Actual – Positive | 0.002 | 0.01 | 25 | 0.20 | .845 |
| Actual – Negative | 0.004 | 0.01 | 25 | 0.57 | .572 |
| Actual – Preference | -0.01 | 0.01 | 25 | -0.69 | .500 |
| Actual – D-Score | 0.004 | 0.01 | 25 | 0.64 | .528 |
| Gut – Positive | 0.003 | 0.01 | 25 | 0.41 | .682 |
| Gut – Negative | 0.005 | 0.01 | 25 | 0.88 | .390 |
| Gut – Preference | -0.01 | 0.01 | 25 | -0.62 | .544 |
| Gut – D-Score | 0.01 | 0.01 | 25 | 0.59 | .560 |
| Positive – Negative | 0.002 | 0.004 | 25 | 0.68 | .501 |
| Positive – Preference | -0.01 | 0.01 | 25 | -0.74 | .467 |
| Positive – D-Score | 0.003 | 0.01 | 25 | 0.31 | .758 |
| Negative – Preference | -0.01 | 0.01 | 25 | -1.03 | .311 |
| Negative – D-Score | 0.0001 | 0.01 | 25 | 0.02 | .984 |
| Preference – D-Score | 0.001 | 0.01 | 25 | 1.05 | .304 |

Table S3.4 contains the mean differences for the main effect of the type of relative prejudice measure. Compare this to Table 11 in the manuscript.

**Table S3.5**. **Post Hoc Tests for Model Comparisons.**

| Comparison | |  |  |  |  |
| --- | --- | --- | --- | --- | --- |
| Model | Mean Difference | *SE* | *df* | *t* | *p* |
| Ideology – Status | -0.03 | 0.05 | 25 | -0.66 | .515 |
| Ideology – Choice | -0.09 | 0.05 | 25 | -1.84 | .078 |
| Ideology – Ideology + Status + Choice | 0.001 | 0.05 | 25 | 0.02 | .981 |
| Ideology – Null | -0.02 | 0.05 | 25 | -0.44 | .667 |
| Status – Choice | -0.06 | 0.05 | 25 | -1.18 | .249 |
| Status – Ideology + Status + Choice | 0.03 | 0.05 | 25 | 0.68 | .500 |
| Status – Null | 0.01 | 0.05 | 25 | 0.22 | .824 |
| Choice – Ideology + Status + Choice | 0.09 | 0.05 | 25 | 1.86 | .074 |
| Choice – Null | 0.07 | 0.05 | 25 | 1.40 | .172 |
| Ideology + Status + Choice – Null | -0.02 | 0.05 | 25 | -0.46 | .650 |

Table S3.5 contains the mean differences for the main effect of model type*.* Compare this to Table 12 in the manuscript.

**Note**: The main effects of model and measure were not significant in the relative measures analyses with the controls removed (which aligns with the analyses with the controls included). The model x measure interaction was not significant either. Our Stage 1 manuscript indicated that we will include demographic controls in our analyses, so we describe those results in the manuscript. For readers interested in the post hoc tests, the data and code for the Study 2 analyses are in the “Stage 2 Data,” and “Stage 2 Code” folders, respectively, on the project’s OSF page: <https://osf.io/buwp7/?view_only=3905d5d1d54b4a499483c03a089b9f6e>. The code with the demographic controls removed is in the “Appendices” folder.
