# Supplementary material for: Registered report: Stress testing predictive models of ideological prejudice
Source: PLoS One. 2025 Oct 13;20(10):e0334152. doi: 10.1371/journal.pone.0334152 (PMC12517488; doi:10.1371/journal.pone.0334152)

**S4 Appendix**

**Study 1 Exploratory Analyses – Mixed ANOVA with Explicitly Political Groups (see lines 1653 -1708 in the Study 1 code)**


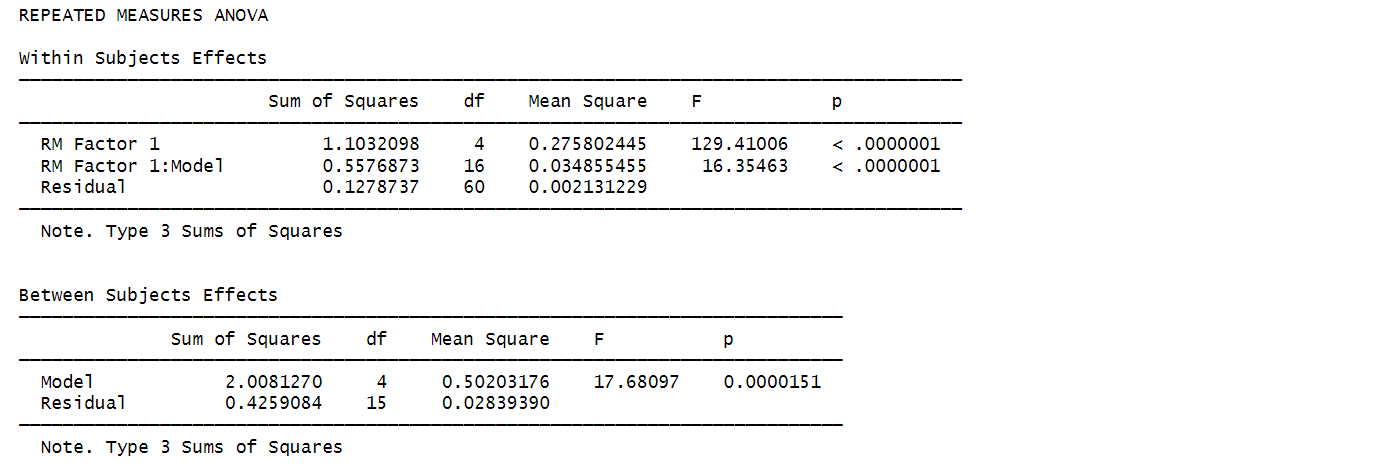


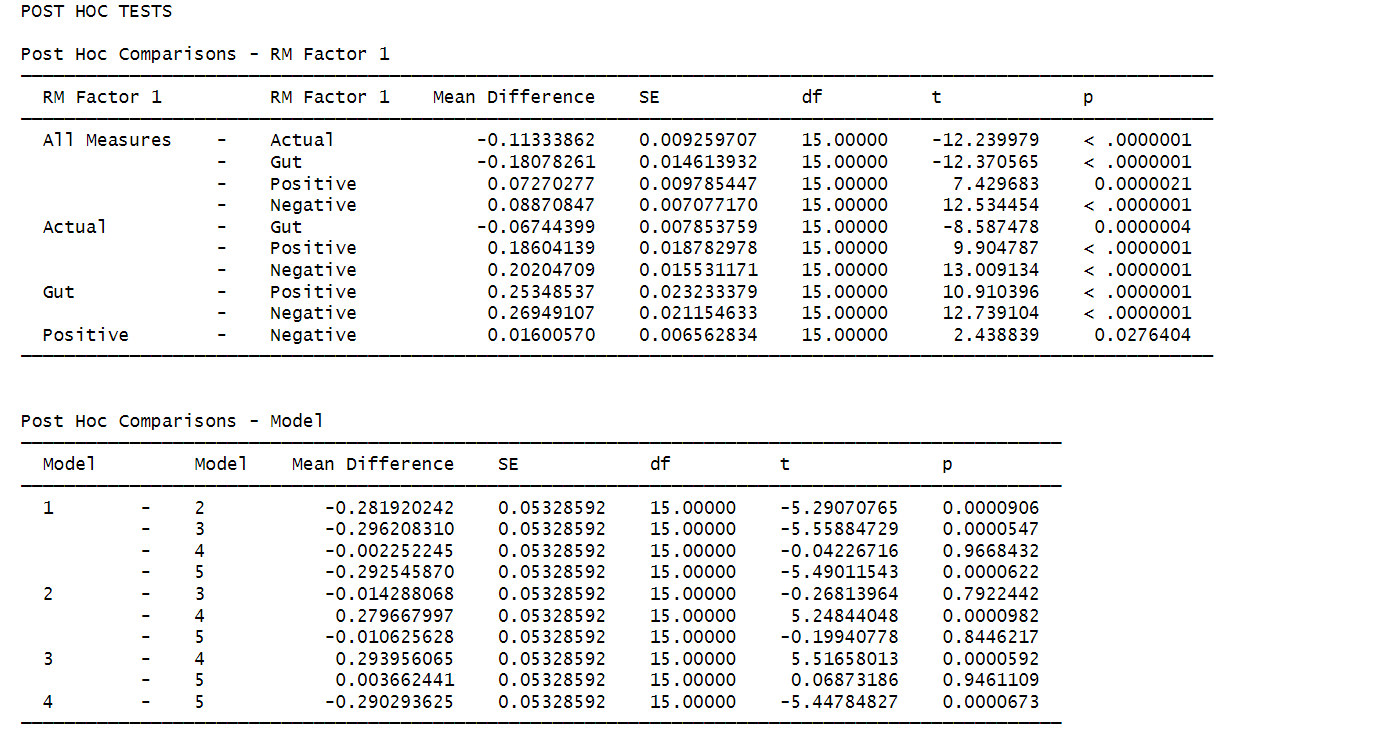


**Study 1 Exploratory Analyses – Mixed ANOVA with All Other Groups (see lines 1710 - 1765 in the Study 1 code)**


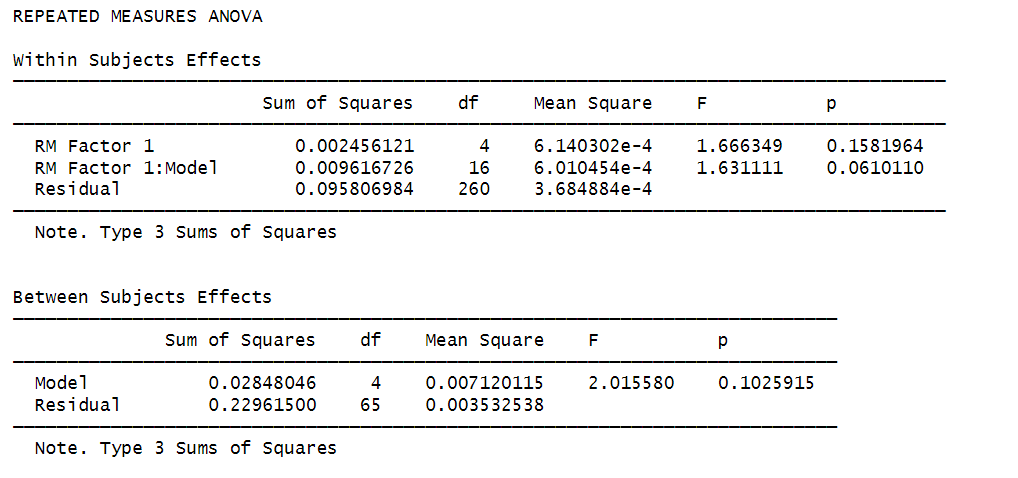


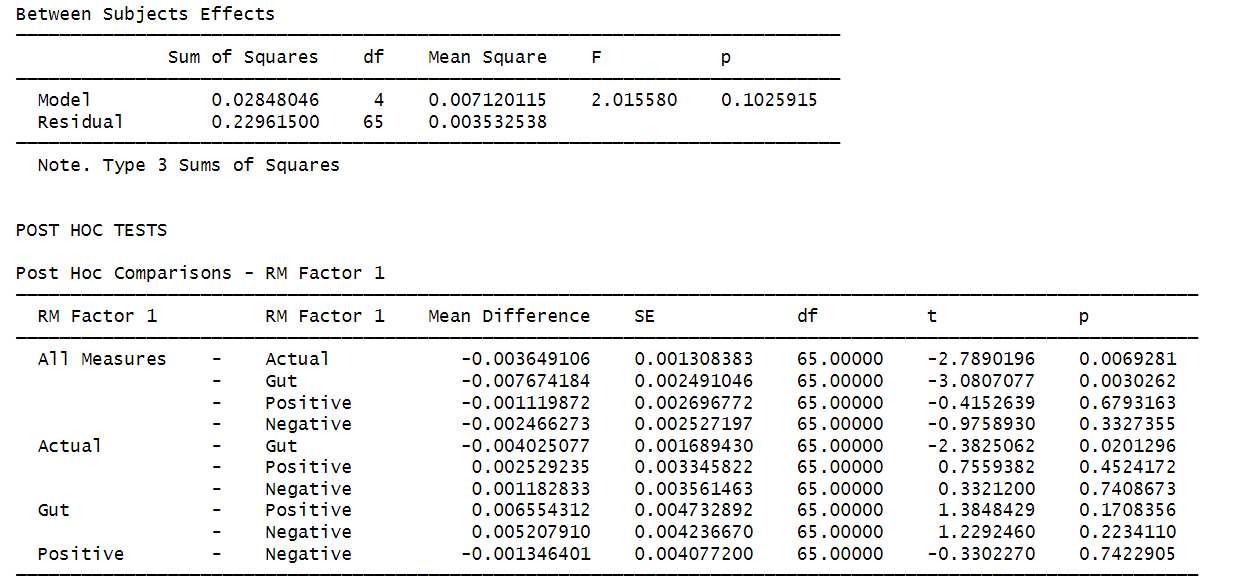

Supplement: S4 Appendix — This supplemental file includes the results for the exploratory mixed ANOVAs for the explicitly political groups and the other 14 groups. (DOCX) [file pone.0334152.s004.docx]
