# Supplementary material for: Registered report: Stress testing predictive models of ideological prejudice
Source: PLoS One. 2025 Oct 13;20(10):e0334152. doi: 10.1371/journal.pone.0334152 (PMC12517488; doi:10.1371/journal.pone.0334152)
Supplement: S5 Appendix — This supplemental file includes the results of the Study 2 analyses with the explicit and implicit relative measures separated. (DOCX) [file pone.0334152.s005.docx]

**S5 Appendix**

**Study 2 Models With Explicit and Implicit Measures Separated**

**Table S5.1 Predictive Equations Generated from the Training Data When Implicit Measures are Removed**

| **Model** **Name** | **Theoretical Implication** | **Model** | **MSE (SD) Estimate Across Outcomes** | **MSE (SD) for Actual Prejudice Difference Outcome** | **MSE (SD) for Gut Prejudice Difference Outcome** | **MSE (SD) for Positive Prejudice Difference Outcome** | **MSE (SD) for Negative Prejudice Difference Outcome** | **MSE (SD) for Preference Difference Outcome** | **BIC with Random Slopes Removed** | **BIC with Random Intercept Removed** | **BIC with Correlation Between Random Slope and Intercept Removed** |
| --- | --- | --- | --- | --- | --- | --- | --- | --- | --- | --- | --- |
| ideology-only | Ideological differences explain ideology-prejudice association | ŷ = 0.063 + 1.140(ideology) | .01 (.01) | .01 (.01) | .01 (.01) | .04 (.04) | .06 (.07) | .02 (.03) | 27332.05 | 21274.61 | 21284.90 |
| status-only | Status differences explain ideology-prejudice association | ŷ = -0.202 – 0.156(status) | .06 (.06) | .04 (.05) | .07 (.08) | .03 (.03) | .04 (.06) | .08 (.10) | 26684.71 | 21580.45 | 26695.01 |
| choice-only | Choice differences explain ideology-prejudice association | ŷ = -0.249 – 0.820(choice) | .05 (.06) | .04 (.04) | .05 (.07) | .03 (.03) | .05 (.06) | .07 (.09) | 27811.61 | 22537.02 | 27821.90 |
| ideology, status, and choice | A combination of group characteristics explains ideology-prejudice association | ŷ = 0.231 + 1.431(ideology) –0.011(status) + 1.238(choice) | .01 (.01) | .02 (.02) | .01 (.02) | .05 (.06) | .07 (.10) | .02 (.02) | 26024.18 | 20019.84 | 20053.60 |
| null | Group characteristics do not explain ideology-prejudice association | ŷ = 0 | .17 (.25) | .13 (.18) | .18 (.26) | .05 (.07) | .04 (.04) | .23 (.33) | **-** | **-** | **-** |

Table S5.1 includes the predictive equations when the implicit prejudice measure is removed and Bayesian Information Criterion (BIC) statistics for the models when random slopes, random intercepts, and the correlation between random slopes and intercepts are removed. Overall, the models with the random intercept removed had the lowest BIC values, so we used these models to generate the predictive equations.

Below are the results of the repeated measures ANOVA for only the explicit measures.


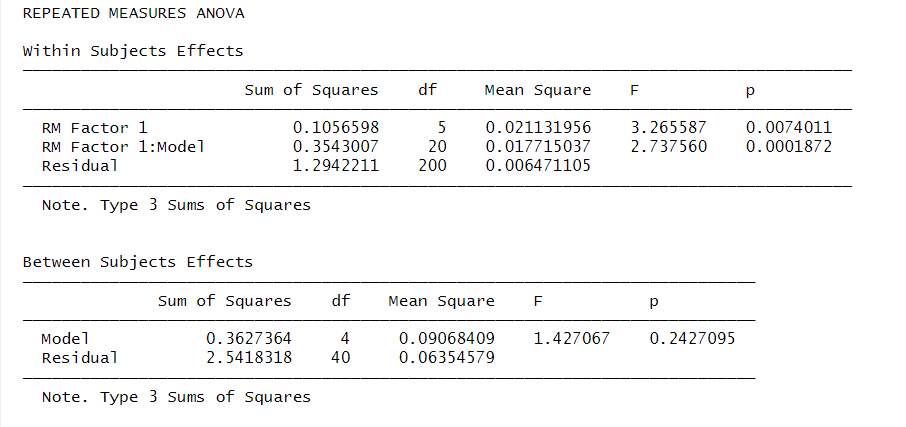


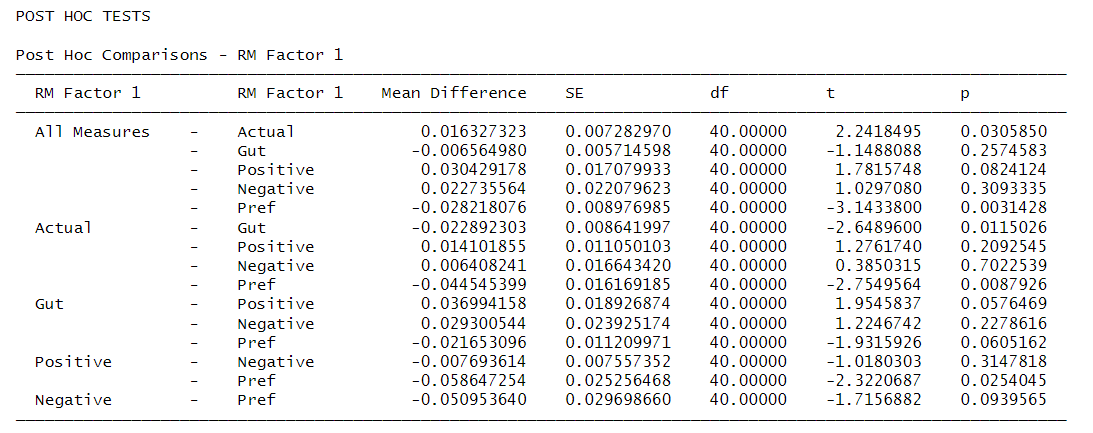


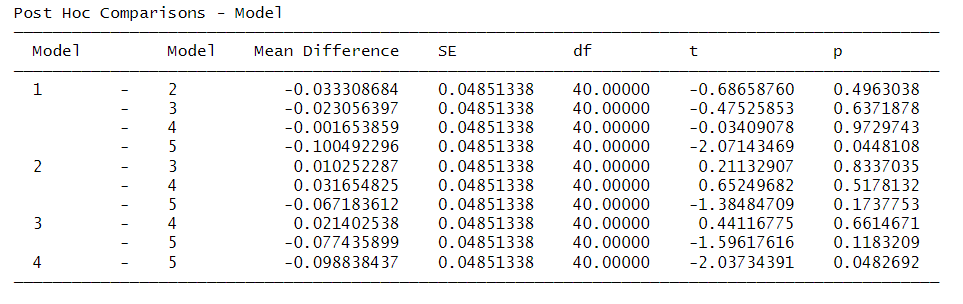


**Table S5.2 Predictive Equations Generated from the Training Data With D_score_xy Measure**

| **Model** **Name** | **Theoretical Implication** | **Model** | **MSE (SD) for D-Score Difference Outcome** | **BIC with Random Slopes Removed** | **BIC with Random Intercept Removed** | **BIC with Correlation Between Random Slope and Intercept Removed** |
| --- | --- | --- | --- | --- | --- | --- |
| ideology-only | Ideological differences explain ideology-prejudice association | ŷ = 0.007 + 0.359(ideology) | .002 (.002) | -5815.88 | -5794.34 | -5806.97 |
| status-only | Status differences explain ideology-prejudice association | ŷ = -0.032 + 0.265(status) | .001 (.001) | -5965.00 | -5942.76 | -5956.09 |
| choice-only | Choice differences explain ideology-prejudice association | ŷ = -0.090 – 0.176(choice) | .002 (.003) | -7129.40 | -7113.83 | -7120.49 |
| ideology, status, and choice | A combination of group characteristics explains ideology-prejudice association | ŷ = 0.043 + 0.370(ideology) –0.090(status) + 0.212(choice) | .001 (.001) | -7708.68 | -7690.63 | -7666.27 |
| null | Group characteristics do not explain ideology-prejudice association | ŷ = 0 | .01 (.01) | **-** | **-** | **-** |

Table S5.2 includes the predictive equations only including the implicit prejudice measure and Bayesian Information Criterion (BIC) statistics for the models when random slopes, random intercepts, and the correlation between random slopes and intercepts are removed. Overall, the models with the random slopes removed had the lowest BIC values, so we used these models to generate the predictive equations.

Below are the results for only the implicit measure (we could not do a repeated measures ANOVA here because there is only one measure type).

**
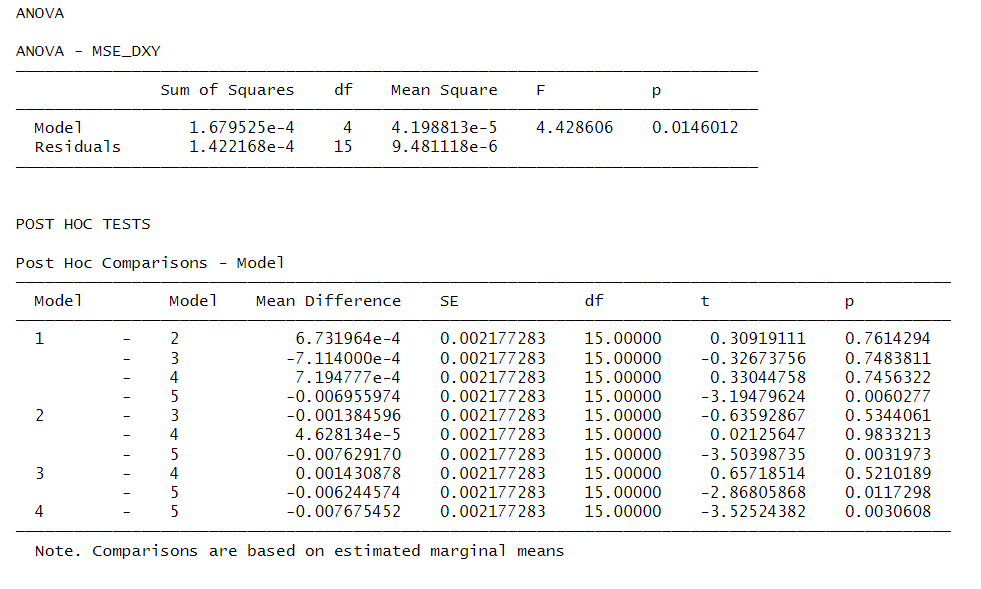
**
