# Supplementary material for: Registered report: Stress testing predictive models of ideological prejudice
Source: PLoS One. 2025 Oct 13;20(10):e0334152. doi: 10.1371/journal.pone.0334152 (PMC12517488; doi:10.1371/journal.pone.0334152)
Supplement: Attachment — Submitted filename: Reviewer Response Letter.docx [file pone.0334152.s007.docx]

**Editor Comments**

Dear Dr. Thompson,

Thank you for submitting your manuscript to PLOS ONE. After careful consideration, we feel that it has merit but does not fully meet PLOS ONE’s publication criteria as it currently stands. Therefore, we invite you to submit a revised version of the manuscript that addresses the points raised during the review process.

As you will find in the reviewer comments, both reviewers find merit in the research presented and both agree that the data may provide a useful contribution to the existing literature. However, both reviewers have concerns that we would like to see addressed in a final version of the paper. Because you have already published a Registered Report Protocol, you will likely need to address these comments to the best of your ability in the Discussion of your paper. Note that Reviewer 1 defers to me in a comment regarding the use of p values in addition to model fit. I recommend that you defer to the planned analyses as indicated in your published protocol (which includes the use of p values). Please feel free to reach out to me if you have any questions regarding the comments; I know it can get tricky when aspects of the paper are already published. 

*We thank the editor for his work on our manuscript. We have addressed the reviewers’ concerns primarily in the discussion and conclusion sections (but we have added a few clarifications in the introduction, see page 3). Additionally, we have retained our use of p-values to maintain consistency with our Stage 1 manuscript.*

*We wanted to mention that in double-checking our code, we noticed that the values for status and choice for Black people were switched by mistake. We have re-run our analyses and found the same pattern of results. The values in the manuscript have been updated to reflect this, and they have changed very slightly. We apologize for this error, and we have double-checked the other values, and they are correct.*

*Please note, as requested in the decision email, we have added two new references. To strengthen our discussion, we have added the references: Koch et al., 2016 and Yustisia et al., 2025.*

Please submit your revised manuscript by Sep 05 2025 11:59PM. If you will need more time than this to complete your revisions, please reply to this message or contact the journal office at [plosone@plos.org](mailto:plosone@plos.org). When you're ready to submit your revision, log on to <https://www.editorialmanager.com/pone/> and select the 'Submissions Needing Revision' folder to locate your manuscript file.

Please include the following items when submitting your revised manuscript:

- A rebuttal letter that responds to each point raised by the academic editor and reviewer(s). You should upload this letter as a separate file labeled 'Response to Reviewers'.
- A marked-up copy of your manuscript that highlights changes made to the original version. You should upload this as a separate file labeled 'Revised Manuscript with Track Changes'.
- An unmarked version of your revised paper without tracked changes. You should upload this as a separate file labeled 'Manuscript'.

If you would like to make changes to your financial disclosure, please include your updated statement in your cover letter. Guidelines for resubmitting your figure files are available below the reviewer comments at the end of this letter.

If applicable, we recommend that you deposit your laboratory protocols in protocols.io to enhance the reproducibility of your results. Protocols.io assigns your protocol its own identifier (DOI) so that it can be cited independently in the future. For instructions see: [https://journals.plos.org/plosone/s/submission-guidelines#loc-laboratory-protocols](https://nam02.safelinks.protection.outlook.com/?url=https%3A%2F%2Ftrack.editorialmanager.com%2FCL0%2Fhttps%3A%252F%252Fjournals.plos.org%252Fplosone%252Fs%252Fsubmission-guidelines%2523loc-laboratory-protocols%2F1%2F010f01983238dbf3-4b066f71-f05c-4596-bf39-49d85eaa547d-000000%2Fw-u2lW7k2SkOqYnb0JxsqV22zL3jQm7GCer6Lz3HYXY%3D220&data=05%7C02%7Cjordanthomps2021%40fau.edu%7C9e9d9a67522648880d4408ddc92b3f65%7C63c3c9c1e824413fb4352f0cabb2828f%7C0%7C0%7C638887909769589057%7CUnknown%7CTWFpbGZsb3d8eyJFbXB0eU1hcGkiOnRydWUsIlYiOiIwLjAuMDAwMCIsIlAiOiJXaW4zMiIsIkFOIjoiTWFpbCIsIldUIjoyfQ%3D%3D%7C0%7C%7C%7C&sdata=fMGRXFpGX9mOPUXNb91k0cDxJWgcnxVWd6YdRjEcG3Q%3D&reserved=0). Additionally, PLOS ONE offers an option for publishing peer-reviewed Lab Protocol articles, which describe protocols hosted on protocols.io. Read more information on sharing protocols at [https://plos.org/protocols?utm_medium=editorial-email&utm_source=authorletters&utm_campaign=protocols](https://nam02.safelinks.protection.outlook.com/?url=https%3A%2F%2Ftrack.editorialmanager.com%2FCL0%2Fhttps%3A%252F%252Fplos.org%252Fprotocols%253Futm_medium%3Deditorial-email%2526utm_source%3Dauthorletters%2526utm_campaign%3Dprotocols%2F1%2F010f01983238dbf3-4b066f71-f05c-4596-bf39-49d85eaa547d-000000%2FhHGWf-VBdxKElwQiPRXjOxka8fCsDDaEj8-MV7zVnSE%3D220&data=05%7C02%7Cjordanthomps2021%40fau.edu%7C9e9d9a67522648880d4408ddc92b3f65%7C63c3c9c1e824413fb4352f0cabb2828f%7C0%7C0%7C638887909769611798%7CUnknown%7CTWFpbGZsb3d8eyJFbXB0eU1hcGkiOnRydWUsIlYiOiIwLjAuMDAwMCIsIlAiOiJXaW4zMiIsIkFOIjoiTWFpbCIsIldUIjoyfQ%3D%3D%7C0%7C%7C%7C&sdata=IDQkzTs7lFc%2F77NY19tJu5bF6tdp2n6wkIq48yzDSio%3D&reserved=0).

We look forward to receiving your revised manuscript.

Kind regards,

Corey Cook
Academic Editor
PLOS ONE

Journal Requirements:

When submitting your revision, we need you to address these additional requirements.

1. Please ensure that your manuscript meets PLOS ONE's style requirements, including those for file naming. The PLOS ONE style templates can be found at

<https://journals.plos.org/plosone/s/file?id=wjVg/PLOSOne_formatting_sample_main_body.pdf> and

[https://journals.plos.org/plosone/s/file?id=ba62/PLOSOne_formatting_sample_title_authors_affiliations.pdf](https://nam02.safelinks.protection.outlook.com/?url=https%3A%2F%2Ftrack.editorialmanager.com%2FCL0%2Fhttps%3A%252F%252Fjournals.plos.org%252Fplosone%252Fs%252Ffile%253Fid%3Dba62%252FPLOSOne_formatting_sample_title_authors_affiliations.pdf%2F1%2F010f01983238dbf3-4b066f71-f05c-4596-bf39-49d85eaa547d-000000%2FS94rYwwBj8FUXUHDagDidaS04Erl2T3TvJQppUfXDQw%3D220&data=05%7C02%7Cjordanthomps2021%40fau.edu%7C9e9d9a67522648880d4408ddc92b3f65%7C63c3c9c1e824413fb4352f0cabb2828f%7C0%7C0%7C638887909769629014%7CUnknown%7CTWFpbGZsb3d8eyJFbXB0eU1hcGkiOnRydWUsIlYiOiIwLjAuMDAwMCIsIlAiOiJXaW4zMiIsIkFOIjoiTWFpbCIsIldUIjoyfQ%3D%3D%7C0%7C%7C%7C&sdata=17POB%2B%2BiesPAOEV2q4ggSS09ZN89ziksn7DkYg7B1iw%3D&reserved=0)

2. In your cover letter, please confirm that the research you have described in your manuscript, including participant recruitment, data collection, modification, or processing, has not started and will not start until after your paper has been accepted to the journal (assuming data need to be collected or participants recruited specifically for your study). In order to proceed with your submission, you must provide confirmation.

3. Please review your reference list to ensure that it is complete and correct. If you have cited papers that have been retracted, please include the rationale for doing so in the manuscript text, or remove these references and replace them with relevant current references. Any changes to the reference list should be mentioned in the rebuttal letter that accompanies your revised manuscript. If you need to cite a retracted article, indicate the article’s retracted status in the References list and also include a citation and full reference for the retraction notice.

**General Response From the Authors**

*We thank the editor and reviewers for their close attention to our manuscript. We respond to each point below. Reviewer comments are in plain text, and our responses are in italics. We also include the relevant sections of the paper in quotes for easy reading. These are in quotes, in italics, and with bullet points.*

**Reviewer 1**

This was a paper extending some previous work building predictive models of prejudice toward various groups. I very much support iterative progression of these more formal modeling approaches, and I thought the adaptation to relative differences, another very common operationalization of prejudice in the field, was an important extension. Generally the authors find additional support for the ideology model over some other specifications. Thoughts and concerns follow:

Major concerns:
Because this paper is basically extending the Brandt 2017 model, some of these questions and critiques necessarily apply to that work as well. It's a little odd because that work is published and established, but still think it makes sense for the authors to engage with some of the questions in the present work.

-One thing I've always wondered with these models is about construct validity, or how much this "rating of ideology" is capturing the same or similar latent construct of prejudice. On their face, certainly distinct to me. But to the extent this variable is capturing "the extent to which i see these various groups on my team or not", it's less surprising to find such a strong link with how much I like those groups.

*We thank the reviewer for bringing up this point. We can see where the reviewer is coming from. However, we do not think that these ideology ratings are capturing the latent concept of prejudice. Our models include two instances of “ideology” – perceived group ideology (and/or status and choice for the other models) and participant ideology. The perception of groups’ ideological leanings is largely consistent and stable (our values were similar to Brandt, 2017 and Brandt & Crawford, 2016). The literature also shows that people have a consensus about a groups’ perceived ideology and agency/competence (similar to status; Koch et al., 2020). In contrast, perceived warmth (closer to our prejudice measure) is not agreed upon (Koch et al., 2020). If perceived ideology and prejudice were tapping into the same latent construct, they should be similarly consensually perceived. As the reviewer alludes to, our measures are highly face-valid, and are commonly used across literatures (e.g., Crawford et al., 2017, Jost, 2006; Jost, 2017). The constructs of one’s ideology and one’s prejudice toward certain groups are related, but they do not completely overlap. The ideological values used in our equations are related to the stable perceptions of groups’ ideologies as opposed to individual participants’ ideological positions.*

*We would also like to point out that the measures of status and choice are much less likely to have the type of shortcoming the reviewer brings up. Our study is also relevant to these perceptions as it shows that these group perceptions do not help predict the ideology-prejudice association. This is notable as theories in social and political psychology suggest that the ideology-prejudice association might be better explained by these variables. We show that not only is it not better explained, it is not explained by status and choice.*

-A related concern is that these models depend on ratings on a bipolar liberal to conservative scale. Such a scale would just not work in the majority of countries in the world in which the political spectrum can't be reduced to one dimension. If our goal is purely non-explanation based prediction in the US, that's totally fine of course. But makes it more challenging to argue this is a causal model of the more universal force of prejudice, or it would have to be tweaked in some way. I guess this critique hinges on how the authors are interpreting this model, and I'd like to see that spelled out.
I've grouped these two together because they have perhaps been engaged with by the authors elsewhere, and I think could be handled here by some mention in the General Discussion.

*This is a good point. We have added a discussion of the generalizability of our results in the Future Directions section (see pages 48-49).*

- *“For both studies, it is useful to consider how the results might generalize outside of the United States. The idea behind the models is that ideological similarity and dissimilarity help explain the ideology-prejudice association across a wide range of target groups. When working in other contexts, it is necessary to use measures of ideology and groups that are relevant for that context. For example, Koch and colleagues’ [39] methods for eliciting important target groups in different cultural contexts can be used to generate the relevant groups for the context. Similarly, a consultation with local experts and the relevant research literature can identify the most important ideological dimensions in the context. Although the liberal-conservative dimension may not be the most relevant outside of the US, alternatives like the left-right dimension, progressive-traditional dimension, or a secular-religious dimension appear in other country contexts (e.g., [40]). With context-relevant groups and ideology measures identified, scholars can follow the methods we’ve used here to develop and test the relevant models in alternative contexts. Although the precise ideological dimension and groups might differ, we could expect that the ideological similarity and dissimilarity of groups would be a relevant factor in explaining the ideology-prejudice association.”*

-Regarding the modeling, I found the use of demographic controls conceptually problematic. If the goal is to predict prejudice from ideology, status, choice, and anything else, why should these be adjusted for the demographics of the participant? To adjust for gender, for example, is to say that men are just inherently more prejudiced toward xyz groups in a way that can't be explained or captured by other psychological variables, and I just don't believe that to be true. Plus it changes the very spare model (ideology) to be a lot more conceptually clunky, like who knows what other psychological baggage is being captured by "man" and "educated", but my guess is quite a bit. How much are results contingent on the inclusion of these demographic controls?

*Thank you for this question. To address this, we ran the analyses in Study 1 and Study 2 without the demographic controls (see S3 Appendix and pages 27 and 40 of the manuscript). Regarding our inclusion of demographic controls, we included them for two reasons. First, to conceptually capture the relationship between ideology and prejudice (as opposed to predicting prejudice) in isolation over and above prejudices potentially related to participant traits (e.g., Black participants liking Black people and vice-versa for White participants). Second, Brandt (2017) included demographic controls, and our intention in Study 1 was to replicate his model testing process as closely as possible. We found the same results patterns with and without the demographic controls.*

- *Page 27: “For the curious reader, we also ran the Study 1 analyses without the demographic control variables and found the same pattern of results (see S3 Appendix). As our Stage 1 report was accepted with the inclusion of controls in our analysis plan, we do not interpret those results further in-text.”*
- *Page 40: “As we did in Study 1, we also ran the Study 2 analyses without the demographic control variables and found the same pattern of results (see S3 Appendix). We interpreted the analyses with the controls here, as this is consistent with our Stage 1 manuscript, and our intention in Study 2 is to run analyses that parallel Study 1.”*

-My largest and perhaps controversial philosophical concern was the use of null hypothesis testing for model selection, and looking at p-values as evidence. I just didn't find it appropriate for the goals here. Models with the lowest average MSE simply fit the data best, regardless of whether they fit "significantly" better than other models with higher MSEs. Data science world would focus only on model fit, and this feels like a psych/data science mashup, employing both frameworks for evidence simultaneously. I'd defer to the editor, but I wouldn't be averse to cutting the p-value framework from the paper entirely, and relying only on model fit to determine the best models (and it would result in an identical conclusion from the line already being walked in the general discussion).

*We thank the reviewer for raising this concern. In the Stage 1 manuscript, we discussed using p-values as a means of determining the model with the best fit. The editor has suggested that we use p-values to remain consistent with the Stage 1 plan, so we have retained our use of p-values, although we do acknowledge the controversies surrounding using them (e.g., Wasserstein & Lazar, 2016). To augment our MSE/p-value interpretations, we ran correlations between the predicted and observed values for each model in both studies (see Table 1, page 7, and Table 11, page 36 for their additions to this manuscript). In both studies, the ideology-only (r = .97, .92) and ideology, status, and choice models (r = .96, .92) had higher correlation coefficients than the status-only (r = .45, .43) and choice-only (r = .17, .62) models.*

-What is an "ideologically relevant group" exactly? There needs to be a clear definition for this, rather than just sounds ideologically relevant to the authors and readers. I'm sympathetic in some respects, yes, those groups certainly seem ideologically relevant. But some of the groups not included in this bundle also seem ideologically relevant, and when I start squinting, many of these groups seem like they could also be ideologically relevant. A clear definition would sort this out, especially because the authors tenatively posit on page 25 that prediction success is better for these groups. Without some explanation, feel this risks being a "just so" story.

*We appreciate the reviewer’s request for clarification. We have added a clearer explanation for which groups can be considered “ideologically-relevant” on page 28. We also changed the term to “explicitly political groups.” The groups we flag in our discussion of the results are explicitly based on ideological beliefs and political identities. We now note this.*

- *“Although the patterns in our planned analyses replicated Brandt [1], we explored whether our models were more predictive of the ideology-prejudice association for explicitly political groups. This seemed relevant to us given the results of the relative measures models reported below (i.e., results were less strong in the subset of measures that did not include explicitly political groups). While many of the included groups can be considered political to some extent, some groups are explicitly political and tied to liberal-conservative politics in the United States (i.e., liberals, conservatives, Democrats, Republicans).”*

-On page 29, the authors mention they combined explicit and reaction time measures. What does this mean? How were they combined. Some folks consider implicit vs. explicit measures of prejudice quite distinct, so this feels odd.

*This is an important clarification. We have updated our description on page 33. To further clarify, we used all the implicit and explicit prejudice measures available in the data set. In order to run our multilevel models, we turned the data long (i.e., such that each participant has multiple rows organized by prejudice measure). When we turned the data long, we created our prejudice outcome variable, which represents both the explicit and implicit prejudice measures(see lines 268-282 in the Study 2 code). In the context of our multilevel models, this means the measures were not combined per se but were nested within participants in separate rows. Given the moderate correlations (i.e., ranging from .35 - .45) between the explicit and implicit measures, this seems justified as these correlations suggest the relationship between these measures is non-trivial.*

*At the same time, we do see the reviewer’s concern. To address concerns about combining the measures, we ran additional analyses with the explicit and implicit measures separated. The resulting predictive equations were different (see the S5 Appendix), but neither found any difference between the ideology, status or choice models (there is some limited evidence that all models did better than the null model). However, given that we wrote in our Stage 1 manuscript that we would combine the measures, we have decided to keep this in the manuscript. We also note our analyses with the measures separated on pages 43-44 and recommend that future researchers could test if separating the measures helps better predict the size and direction of the ideology-prejudice association (page 45).*

- *Page 33: “In our analyses, we used both the explicit relative measures and the reaction time outcomes because they are measures of comparative prejudice* *(they were also moderately correlated, see Table 3). Specifically, these measures both involve making a choice to indicate preference for one group over another. In the context of our multilevel models, this means each measure was nested within participants in separate rows.”*
- *Pages 43-44: “**Per a reviewer’s suggestion, we also ran the Study 2 analyses with the explicit and implicit relative measures separated. Although the explicit and implicit measures were moderately correlated (ranging from .35 - .45, see Table 3), these correlations indicate potential differences between the measures. For full results, see the S5 Appendix. Most importantly, the effect of model type was not significant, F(4, 40) = 1.43, p = .243, which indicates that there were no significant differences in the predictive accuracy of the models. The interaction between measure type and model was significant, F(5, 200) = 2.74, p < .001. The interaction was largely driven by significant differences that indicated that the experimental models had better predictive ability than the null model for some of the measures, but there were no differences between the alternative models. For the implicit measures, the effect of model type was significant, F(4,15) = 4.43, p = .015. The post-hoc tests revealed that the only significant differences between the models were between the null model and the alternative models, such that the null model performed worse than all other models. In the case of the pre-planned Study 2 analyses, and the analyses described here, we found that there were no significant differences in the models’ predictive abilities (besides comparisons with the null model). Because we wrote in our Stage 1 manuscript that we would combine the measures, we do not interpret them strongly here.”*
- *Page 45: “**Additionally, we ran exploratory analyses to examine how the separation of the explicit and implicit relative measures impacted the results. We did not state that we would do these additional analyses in our Stage 1 manuscript, but we felt it was an important set of analyses to consider given the moderate correlations between the explicit and implicit measures. We do not make strong claims about the results of these analyses, as they were not pre-registered or a part of the initial Stage 1 manuscript. Future researchers could test the differences between explicit and implicit measures in future model-building attempts. It is possible that explicit and implicit measures require different model-building strategies.”*

Minor concerns:
-Intro of 2nd para- the prejudice literature has offered far more than just 3 predictors of prejudice. Maybe can revise this sentence to suggest Brandt identified three in his models. The entire field has prob put out at least 50.

*We thank the reviewer for raising this point. We have revised the sentence to clarify that we are referring to the predictors Brandt (2017) focused on (page 3). Additionally, we updated the statement to make it clearer that we are predicting the ideology-prejudice association, as opposed to predicting prejudice (for which we agree there are dozens of proposed predictors).*

- *“In effort to forecast prejudice toward various groups, Brandt [1] focused on three main predictors of the ideology-prejudice association: the ideological position of a group, the group’s status, and the extent to which group membership is a choice.”*

-A line in the General Discussion mentions this is the "first time" predictive models were built for relative prejudice. While this is possibly true for the Brandt ideological prejudice models, it's not in general. Hehman & Neel psych review recently had some predictive models of prejudice with relative measures (and non-relative measures, a nice parallel to the current work and Brandt 2017). In general, I don't always find lines in papers noting "first" are so important, since that's somewhat obviated as the point of the paper, and could just be adjusted or trimmed.

*We appreciate the reviewer’s attention to detail. We have removed our claim of being the first to use relative measures in building models to predict the ideology-prejudice association.*

-Lot of measures, would be great to have a correlation table between everything somewhere, apologies if I missed this.

*We thank the reviewer for this suggestion. We have added a correlation table on page 18.*

**Reviewer 2**

This paper presents a “stress test” of models for predicting the ideology-prejudice association, comparing models that incorporate the perceived ideology, status, and choice in group membership of various target groups. I have focused this review on the results, conclusions, and adherence to the initial registered report protocol, given that the methods and research question have been reviewed previously. Overall, the manuscript adheres to the procedures and analyses described in the protocol, and exploratory analyses are clearly marked. The authors were careful not to place too much weight on exploratory analyses relative to pre-planned analyses. However, my main concern with the manuscript is that the results of one of the exploratory analyses drastically alter the overall conclusions that I believe can be drawn from the manuscript. Despite this analysis not being pre-planned, I believe the changes to the results are sufficiently large that the authors should temper their conclusions in the paper to take them into account. I expand on this point below. If the conclusions and discussion can be revised to better incorporate the results of this analysis, I believe this manuscript will be well-suited for publication and provide an important contribution to the literature.

*We thank the reviewer for their positive feedback. We have clarified our interpretations of the results in the discussion and conclusion sections. We detail our changes in response to the points below.*

In my reading, the exploratory analysis reported on page 25 very significantly alters the results and warranted conclusions of the paper. The authors find that when they exclude targets groups that are defined by ideology (e.g., Democrats and Republicans), there are no differences among the predictiveness of the various models (including the null model). This seems highly significant to me because the claim that perceived ideology predicts prejudice is only theoretically meaningful or interesting insofar as it is about groups not explicitly defined by ideology. It seems trivial to say that the biggest predictor of how much Republicans like Democrats (and vice versa) is their perceived ideology – in fact these seem more like control groups than target groups to me. The results of the exploratory analysis indicate that the advantage of the ideology models over the status, choice, and null models is solely due to the inclusion of these groups that are explicitly defined by ideology. However, the authors’ discussion, conclusion, and abstract still make strong statements about the benefit of the ideology model(s) over the other models, which I believe are unwarranted given these results. I believe these conclusions and interpretations should be revised to reflect the fact that ideology provides no better prediction than the other models for the groups that are not defined only by ideology.

*We appreciate the reviewer’s interest in our exploratory analyses, and we agree that future researchers should take these results into consideration. However, as these were exploratory analyses and this paper is based on a Stage 1 pre-accepted method (see comments from the editor), we did not interpret them strongly in our manuscript, and we prioritized interpreting the analyses described in our Stage 1 manuscript. Our interpretation of our exploratory results is that the models’ predictions do not differ much for the groups in our data that are not explicitly political. This is because the models make largely similar predictions for groups who are perceived as more moderate. One of the strengths of this model-building strategy is that as many groups as possible were included to represent as much of the group perception and ideology-prejudice conceptual space as possible. This is a methodological strength because if the models do not differ for some subset of groups and if we only included those groups, we would make the wrong conclusions about the whole sample of groups. By developing our models on a broader range of groups (explicitly political groups included), the models are able to make predictions about the broader population of groups. When groups for which the models make substantially different predictions are removed, the test of the models becomes less stringent. Indeed, by removing such groups, we are creating a situation where the models make highly similar predictions, which is a poor situation for testing between models.*

*We continue to interpret our Study 1 exploratory analyses as exploratory. We’ve clarified how we think about these results on pages 29-30 and expand on our interpretation in the General Discussion on page 46.*

- *Pages 29-30: “We want to acknowledge that the results of our exploratory analyses with explicitly political groups removed suggested that the strength of the ideology-only and ideology, status, and choice models may be attributed to the inclusion of these explicitly political groups. Although we remain cautious in over-interpreting these exploratory analyses, as we chose to do those analyses post-hoc after our Stage 1 manuscript was accepted, we want to discuss two plausible ways to interpret these results. First, these exploratory results could reveal a major limitation of the ideology-only model in that the model only works well when these explicitly political groups are included. Second, these exploratory results could reveal that to fully test and understand models of ideology and prejudice, it is important to use a wide range of groups that represent the broader population of groups in society. For many groups, the models make similar predictions (see the black dots in Fig 1), but their predictive advantages only emerge when including those groups where they make different predictions. To test between models, it is necessary to include groups where the models make different predictions.”*
- *Page 46: “Here, we are placing more weight on the planned analyses described in our Stage 1 manuscript that contain the full range of available target groups. However, our exploratory analyses with explicitly political groups removed indicated that the accuracy of the ideology-only and ideology, status, and choice models may be due to their better predictive ability for these groups compared to the status-only and choice-only models. This is because the models do not make substantially different predictions for many of the other groups in the study. Other groups with strong ideological stereotypes (e.g., religious people, atheists; [1], [39]) were not available in this data, but are groups where the models are likely to make divergent predictions.”*

On a separate note, the logic of the analyses was not always intuitive, and I believe in some places could benefit from additional examples or explanation. At the top of page 20, for example, the authors discuss calculating a predicted ideology-prejudice association for the group “Black people”. Here, it would be very helpful to walk the reader through what each of the values represents – what does the final estimate of -0.49 in this context mean? Further, the authors at times refer to predicting prejudice and at other times to the ideology-prejudice association, and I think this distinction could be clarified and highlighted more. As I understand, the main purpose of the study is to predict the association between ideology and prejudice; however, on page 20, for example, the authors seem to be working with actual prejudice values rather than associations (“we subtracted the actual ratings of prejudice across groups from the estimates from the equations, squared these values, and then found the average”). Additional explanation would be helpful here – why are actual prejudice values rather than associations being predicted here? Finally, at times the distinction between the participant’s ideology and the perceived ideology of the group became confusing, and I would encourage the authors to explicitly state which of the two they are referring to when they use the word “ideology” in order to reduce cognitive load for the reader.

*We appreciate this comment, as it allowed us to clarify our description of how the ideology-prejudice association values should be interpreted. We also realized that we mistakenly used the re-scaled perceived ideology for Black people (0.3612, with a raw value of 36.12 out of 100), instead of the midpoint-centered re-scaled value (-0.1388). We have updated our walkthrough of the calculation and interpretation on page 22. We also went through the manuscript and ensured that we clarified whether we were discussing perceived or participant ideology and ensured that we clarified that we are predicting the ideology-prejudice association.*

- *“For example, referring to Table 1, to calculate the predicted ideology-prejudice association for the target group Black people using the ideology-only model (using Brandt’s [1] original equation and the group ratings from our new data collection), we used this equation, ŷ = 0.022 – 1.420(ideology), and substituted “(ideology)” with -0.1388: ŷ = 0.022 – 1.420(-0.1388). Solving for y, we arrive at the estimate of the ideology-prejudice association, .22. This means for that any group with a perceived ideology of -0.1388 (in our sample of target groups, Black people; the value was midpoint-centered), the model predicts that the association between ideology and prejudice is .22. The interpretation of this estimate depends on how ideology and prejudice is coded. We code participant ideology to range from 0 to 1 (0 = very liberal to 1 = very conservative), and prejudice is coded to range from 0 to 1 (0 = low levels of prejudice to 1 = high levels of prejudice). This means that the model predicts that as we go from the lowest point on the ideology scale (0 = very liberal) to the highest (1 = very conservative), participants are predicted to score higher on the prejudice measure by .22 (or about 22% of the scale range).”*

Below are a few additional minor questions:
1. Could the authors expand on the choice to use mean squared error as a metric to compare models, rather than a metric that takes into account the number of parameters included in the model?

*We thank the reviewer for this request for clarification. We acknowledge that MSEs are a controversial metric for comparing models. In this Stage 2 report, we used MSEs for two primary reasons. First, we wrote in our Stage 1 manuscript that we would use MSEs to compare the models. Second, we wanted to replicate Brandt’s (2017) model-building and comparisons as closely as possible. He used MSEs to compare his models, and obtained similar values to ours (our ideology-only and ideology, status, and choice models both had MSEs of .01, and his models’ MSEs ranged from .01-.04). To reinforce our results, we also calculated correlations between the predicted and observed values for each model (see Table 1, page 7, and Table 11, page 36). In both studies, the ideology-only (r = .97, .92) and ideology, status, and choice models (r = .96, .92) had higher correlation coefficients than the status-only (r = .45, .43) and choice-only (r = .17, .62) models.*

2. Were all the demographic control variables in the models used to get the observed ideology-prejudice associations the same as the ones used by Brandt (2017) to get the predicted ones?

*This is important to clarify. We used the same demographic control variables as Brandt (2017) to obtain the observed values, except income, which had substantial missing data. We clarify this on page 35. Additionally, we ran the analyses with and without the controls and found the same pattern of results (see S3 Appendix).*

- *“Additionally, we controlled for gender with men as the reference group, education with less than a high school education as the reference group, religion with Christians as the reference group, ethnicity with American Indian/Alaskan Native as the reference group and mean-centered age. All variables, except the target characteristic differences, were recoded to range from 0-1 so that the coefficients could be interpreted as the percent of change in the outcome as one goes from the lowest to the highest value in the measure. We used the same control variables as Brandt [1] except for income, which we excluded because of large amounts of missing data. To create the target characteristic difference values, we rescaled the original target characteristic values from 0-1 and then subtracted the traditionally higher-status group’s value from the lower-status group’s value for each pair (e.g., Gay ideology value - Straight ideology value). We used this method because recoding the difference score to range from 0-1 would result in a dependent measure indicating difference versus no difference, but in a non-directional fashion, which would make interpretation difficult.”*
